# Identification of nuclear factor YA6 genes in sorghum and characterization of their involvement in drought tolerance

**DOI:** 10.3389/fpls.2025.1524066

**Published:** 2025-03-19

**Authors:** GuoJiang Wu, ZhenGuo Wang, Yan Li, PinTing Du, XinYu Liu, Jie Hou, Wei Zhou, YaXing Zhou

**Affiliations:** ^1^ Key Laboratory of State Ethnic Affairs Commission of Ecological Agriculture in Horchin Sandy Land, College of Agriculture, Inner Mongolia Minzu University, Tongliao, Inner Mongolia, China; ^2^ Tongliao Academy of Agricultural Science, Tongliao, Inner Mongolia, China

**Keywords:** sorghum, NF-YA gene family, bioinformatics, drought stress, expression spectrum

## Abstract

Nuclear factor Y alpha proteins (NF-YAs) are conserved transcription factor proteins crucial to plant growth and development that exhibit specific responses to biotic and abiotic stresses. Using bioinformatics approaches to investigate the *NF-YA* family in sorghum (*Sorghum bicolor*), we identified nine *SbNF-YA* genes unevenly distributed on four of the 10 sorghum chromosomes. Despite variations in gene structure, all encode proteins have the characteristic CBFB_NFYA domain and other predicted motifs. The secondary structure of SbNF-YA members is predominantly composed of α-helices and random coils. A phylogenetic analysis of NF-YAs of sorghum and other plant species indicated that SbNF-YAs are closely related to NF-YAs from maize (*Zea mays*) and distantly related to those in Arabidopsis (*Arabidopsis thaliana*). A colinearity analysis determined that six of the nine *SbNF-YA* genes arose from segmental duplication events. Transcriptome and RT-qPCR analyses showed that the expression levels of eight of the *SbNF-YA* genes (*SbNF-YA5* being the exception) are responsive to drought stress to varying degrees. Notably, *SbNF-YA1*, *SbNF-YA4*, *SbNF-YA6*, *SbNF-YA8*, and *SbNF-YA9* expression was significantly upregulated under the stress conditions, suggesting that they participate in drought response. When heterologously expressed in Arabidopsis, *SbNF-YA6* conferred greater tolerance of drought stress imposed by treatment with the osmolyte mannitol, with the transgenic Arabidopsis lines showing superior germination rates; longer roots; higher fresh weight; higher activities of the enzymes peroxidase, superoxide dismutase, and catalase; and higher soluble protein and proline contents, compared to the wild type. Additionally, the transgenic Arabidopsis lines accumulated lower levels of hydrogen peroxide, superoxide anion, and malondialdehyde. The expression levels of several drought-responsive genes were elevated in transgenic Arabidopsis seedlings relative to the wild type, indicating that the heterologous expression of *SbNF-YA6* enhances the drought tolerance of Arabidopsis.

## Introduction

Drought represents a major agricultural challenge globally, adversely affecting crop growth and development and posing a persistent threat to human food security. Approximately 80%–95% of plant fresh biomass consists of water, which is crucial for plant growth, development, and metabolism (Brodersen et al., 2019; Abbasi and Abbasi, 2009). In recent years, the increasing global population and climate change have exacerbated the challenge of meeting agricultural demands with available freshwater resources (Fang et al., 2022). Therefore, investigating the mechanisms by which crops respond to drought stress is of paramount importance for enhancing drought tolerance, mitigating the consequences of climate change, and ensuring food security.

Drought stress severely affects plant growth and crop yield (Miguel et al., 2020) by compromising photosynthesis, respiration, nutrient uptake, energy metabolism, and enzyme activity, ultimately leading to lower yield (Bhatt and Srinivasa, 2005). Additionally, drought stress often affects plant osmotic pressure, resulting in the loss of cell turgor, preventing cell expansion and division, and thus slowing plant growth (Karthikeyan et al., 2007). Plants have evolved various strategies that mitigate the effects of drought stress, including morphological, physiological, and biochemical acclimation responses (Ingram and Bartels, 1996; Xiong et al., 2022; Zhu, 2022; Shinozaki et al., 2003; Bohnert et al., 2006). Some of these strategies avoid excessive dehydration stress by increasing water uptake or minimizing water loss, while others protect plant cells from damage due to dehydration (Verslues et al., 2006). Severe drought stress can induce the transcription of genes encoding dehydration-responsive element-binding proteins (DREBs) in plants, which then activate the expression of genes involved in detoxification, water and ion uptake and transport, and chaperone function. Changes in gene expression play essential roles in plant drought stress response. For example, the APETALA2 (AP2)/ETHYLENE-RESPONSE FACTOR (ERF), basic leucine zipper (bZIP), NAC, homeodomain leucine zipper (HD-ZIP), and MYB families of transcription factors have specific functions in drought tolerance in various plant species such as Arabidopsis (*Arabidopsis thaliana*), rice (*Oryza sativa*), and maize (*Zea mays*) (Zhou et al., 2012; Chang, 2019; Bang et al., 2018; Zhang et al., 2004).

Nuclear factor Y (NF-Y) transcription factors are ubiquitous in eukaryotes and are also known as heme activator proteins (HAPs) (Petroni et al., 2012). NF-Ys bind to CCAAT-box motifs in the promoter of their target genes and are therefore also known as CCAAT-binding factors (CBFs) in mammalian cells (Laloum et al., 2013). NF-Ys are a large family comprising NF-YA (CBFB or HAP2), NF-YB (CBFA or HAP3), and NF-YC (CBFC or HAP5) subunits (Nardini et al., 2013). In Arabidopsis, the expression of *NF-YA5* genes is regulated transcriptionally and post-transcriptionally (Li et al., 2008). Overexpression of *NF-Y* in plants can promote plant development processes, such as embryo formation (Kwong et al., 2003), seed germination (Warpeha et al., 2007), flowering time (Kumimoto et al., 2008), primary root elongation (Ballif et al., 2011), photosynthesis (Stephenson et al., 2011), endosperm development (Sun et al., 2014) and photomorphogenesis (Myers et al., 2016), and improve tolerance to drought (Wang et al., 2018; Li et al., 2021), salinity (Wu et al., 2018) and osmotic stress (Feng et al., 2015). NF-YA proteins are involved in multiple aspects of the plant life cycle. In Arabidopsis, NF-YA3 and NF-YA8 mediate cell differentiation and embryo formation through the abscisic acid (ABA) signaling pathway during early embryonic development (Mu et al., 2013). NF-YA1, NF-YA5, NF-YA6, and NF-YA9 are involved in the development of gametes, embryos, and seeds (Mu et al., 2013). *NF-YA5* transcript abundance is regulated by the microRNA miR169, thereby influencing the tolerance of Arabidopsis plants to drought stress (Li et al., 2008). In rice, overexpression of *OsNF-YA7* enhances drought tolerance through an ABA-independent pathway (Lee et al., 2015). In potato (*Solanum tuberosum*), NF-YAs regulate chlorophyll content, stomatal conductance, and photosynthesis in response to drought stress (Li et al., 2021). In soybean (*Glycine max*), GmNF-YC14 forms a heterotrimer with GmNF-YA16 and GmNF-YB2 that activates the PYRABACTIN RESISTANCE 1 (GmPYR1)-mediated ABA signaling pathway, thus regulating stress tolerance (Yu et al., 2021). In maize, ZmNF-YB16 can form a heterotrimer with ZmNF-YC17 and ZmNF-YA1 that binds to the CCAAT box in the promoter of stress response and growth-related genes through the ZmNF-YA1 subunit to regulate their expression (Wang et al., 2018).

Sorghum (*Sorghum bicolor* L. Moench) is a C_4_ crop of the genus *Sorghum* in the Gramineae family. It is the world’s fifth-largest and most prominent grain after wheat (*Triticum aestivum*), rice, maize, and barley (*Hordeum vulgare*) (Pilar et al., 2020). A long-standing cultivated crop, sorghum was first discovered in the Niger River in western Sudan 8,000 years ago (Wendorf et al., 1992). Its subsequent spread and adaptation in Africa and Asia led to the emergence of more diverse populations of sorghum (Harlan and de Wet, 1972). Due to its high yield and ability to grow on barren soil, sorghum is a staple food for millions of people living in the arid and semi-arid regions of Africa and Asia (Hariprasanna and Rakshit, 2016). Sorghum can also be used as animal feed and building materials and as components of fences and brooms (Mundia et al., 2019), making it highly commercially valuable. The sorghum genome (730 Mb) is much smaller than that of maize (2.4 Gb). Sorghum has become a potential model system for high-yielding C_4_ crops (McCormick et al., 2018), and its diploid genome sequence provides a basis for exploring the function of transcription factors in this plant. At present, multiple transcription factor families have been identified and analyzed in sorghum, including the GATA, LIM, NAC, C2H2, GRAS, and WRKY families (Ge et al., 2022; Lu et al., 2023; Jiao et al., 2023; Niu et al., 2022; Yao et al., 2023; Sarkar et al., 2023; Punia et al., 2021; Cui et al., 2022; Fan et al., 2021; Baillo et al., 2020). However, the contribution of SbNF-YAs to drought stress has not been studied.

In this study, we identified the *NF-YA* subunit genes in sorghum and determined their expression patterns under drought stress. We verified these expression patterns by reverse transcription–quantitative PCR (RT-qPCR) analysis. As *SbNF-YA6* expression was upregulated under drought stress, we chose to characterize *SbNF-YA6* by generating transgenic Arabidopsis lines that heterologously express this gene. Arabidopsis lines expressing *SbNF-YA6* were more drought-tolerant throughout their life cycle. This study provides a reference for an in-depth analysis of the biological functions of NF-YA transcription factors in sorghum, along with genetic resources for molecular breeding of drought tolerance.

## Materials and methods

### Identification and basic analysis of the physical and chemical properties of the *SbNF-YA* gene family members

The sequences of the sorghum genome, its encoded proteins, the coding sequences of all genes, and the GFF annotation file were downloaded from the Phytozome database (https://phytozome-next.jgi.doe.gov/). Then, predicted proteins containing the CBFB_NFYA Pfam domain (PF02045) were identified at the Pfam (https://pfam.xfam.org) protein database via a hidden Markov model (HMM) search. The Hmmer 3.0 software was used to search and compare the SbNF-YA protein sequences, and sequences with a threshold below 0.05 or with missing domains were eliminated. The candidate SbNF-YA proteins were also used as a query for a local BLAST search against the Arabidopsis NF-YA proteins downloaded from the TAIR (https://www.arabidopsis.org) website. The results from these two methods were integrated and converted to a non-redundant list. The sequence IDs were submitted to the TBtools software to extract the protein sequences, which were then submitted to the NCBI-CDD search tool to retrieve functional domains. Proteins lacking the CBFB_NFYA Pfam domain were removed, yielding the final list of the SbNF-YA family members. The physicochemical properties and predicted subcellular location of each SbNF-YA member were obtained via the online software Expasy (https://web.expasy.org/protparam/) and WoLF PSORT (https://wolfpsort.hgc.jp/), respectively. The online software SOPMA (http://npsa-pbil.ibcp.fr/cgi-bin/npsa_automat.pl?page=npsa_sopma.html) was used to predict the secondary structure of the SbNF-YA family members, and the TBtools software was used to display the position of *SbNF-YA* family genes on the chromosomes.

### Sequence alignment analysis of conserved motifs and core domains of SbNF-YA family proteins

The structural characteristics of *SbNF-YA* introns and exons were analyzed based on the GFF annotation file of the sorghum genome. The phylogenetic tree of SbNF-YAs was reconstructed by the neighbor-joining (NJ) method and the Poisson model using the MEGA 11.0 software. The bootstrap value was set to 1,000 replicates, with other parameters set to default. The conserved motifs in each SbNF-YA protein were identified using the MEME suite (https://meme-suite.org/meme/doc/meme.html), and the number of motifs was set to 10. Finally, the TBtools software was used to visualize the introns and exons, phylogenetic tree, and conserved motifs of the SbNF-YA family members. The MEGA 11.0 software was used to manually remove some amino acid sequences of the SbNF-YA family members and retain the core amino acid sequence. The ClustalW (https://www.genome.jp/tools-bin/clustalw) online software was used to align the core amino acid sequences, which were saved as an ALN file. The core sequence alignment results were modified using the ESPript 3.0 software.

### Phylogenetic analysis of NF-YA family proteins in sorghum and *Arabidopsis*, rice, maize, soybean, and millet

The genome files, GFF annotation files, and protein files for *Arabidopsis*, rice, maize, soybean, and foxtail millet (*Setaria italica*) were downloaded from the Phytozome database. Multiple amino acid sequence alignment of the NF-YA family members from sorghum and other species was performed using ClustalX (http://www.clustal.org/clustal2/). The phylogenetic tree was reconstructed using the MEGA 11.0 software. The Evolview: Tree View (http://www.evolgenius.info/evolview/#/treeview) online tool was used to modify the phylogenetic tree.

### Replication and colinearity analysis of NF-YA gene in sorghum

The colinearity between the sorghum genome and the Arabidopsis, rice, maize, and millet genomes, and the repetitive sequences between the *SbNF-YA* gene family members, were obtained using the MCScanX software. The TBtools software was used to visualize the colinearity relationship diagram of the *SbNF-YA* gene family within and between species.

### Analysis of *cis*-acting elements in the *SbNF-YA* promoters

The TBtools software was used to extract a 2,000-bp sequence fragment upstream of the transcription start site of each *SbNF-YA* gene. Each sequence was uploaded to PlantCare (http://bioinformatics.psb.ugent.be/webtools/plantcare/html/) to identify regulatory and response *cis*-elements and then to visualize the results.

### Analysis of *SbNF-YA* transcript targeting by microRNAs and protein interaction analysis of SbNF-YAs

The *SbNF-YA* coding sequences were submitted to psRNATtarget (https://www.zhaolab.org/psRNATarget/) to assess whether and which miRNAs are complementary to each sequence. The alignment of the miRNA and the corresponding target transcript was drawn using the Wei Sheng Xin tool (http://www.bioinformatics.com.cn/plot_basic_miRNA_target_network_plot_197). The protein–protein interaction network between the individual SbNF-YA family members was extracted using the STRING database (https://cn.string-db.org/).

### Analysis of the tissue expression patterns of *SbNF-YA* genes

Gene expression data from different tissues of sorghum were downloaded from the ArrayExpress database under accession number E-MTAB-3839 (https://www.ebi.ac.uk/biostudies/arrayexpress). The expression levels for the nine *SbNF-YA* genes for six tissues (floral meristems, flowers, plant embryos, roots, shoots, and vegetative meristems) were extracted and visualized as a heatmap using the TBtools software.

### Transcriptome analysis of *NF-YA* genes under drought stress in sorghum

The published transcriptome data for two sorghum varieties that are tolerant of drought stress at different stages (accession number: GSE128441; Varoquaux et al., 2019) were downloaded from the Gene Expression Omnibus (GEO) database. Of the nearly 400 independent samples collected along a time series from the two varieties, the expression data from leaf samples of the preflowering drought-tolerant sorghum variety RTx430 were extracted. The TBtools software was used to visualize the expression levels of *SbNF-YA* genes before and after flowering.

### RT-qPCR analysis of *NF-YA* genes under drought stress in sorghum

The extremely drought-tolerant sorghum germplasm ‘9704X3’ was used as the experimental material. Full grains of uniform size were selected, and their surface was disinfected by soaking in a 1% (w/v) NaClO solution for 10 min. After being washed with distilled water three times, the disinfected seeds were placed evenly in a Petri dish containing two layers of filter paper soaked with water. The Petri dish was placed in a growth chamber with a 25°C/16°C day/night cycle, under a 16-h light/8-h photoperiod, with a relative humidity of 60%. When the height of germinated seedlings reached 2–3 cm, they were transplanted into a germination box containing vermiculite for culture, with irrigation applied as half-strength Hoagland nutrient solution every 3 days. When the seedlings had grown to the two-leaf stage, the seedlings were thinned to 10 seedlings with similar growth vigor in each box for simulated drought stress treatment imposed by 15% (w/v) polyethylene glycol 6000 (PEG-6000). Samples were collected immediately before the onset of treatment (0 h, CK) and at 6, 12, 24, 48, and 72 h into treatment, with three replicates per group. The samples were frozen in liquid nitrogen and stored at −80°C for later use.

Total RNA was extracted from the leaves using a TriGene Reagent kit (GenStar, Beijing, China). The concentration and purity of the RNA were determined on a Nano-500 micro-spectrophotometer. First-strand cDNA was synthesized using a StarScript Pro All-in-one RT Mix with gDNA Remover reverse transcription kit (GenStar, Beijing, China). *SbNF-YA* genes were amplified using 2× RealStar Fast SYBR qPCR Mix (GenStar, Beijing, China). Each qPCR tube contained 10 μL of 2× RealStar Fast SYBR qPCR Mix, 0.5 μL each of the upstream and downstream primers, 1 μL of first-strand cDNA, and 8 μL of ddH_2_O in 20-μL total volume. The qPCR amplification program was as follows: 95°C pre-denaturation for 2 min, then 40 cycles of 95°C denaturation for 15 s, 58°C annealing for 30 s, and 72°C extension for 30 s. The internal reference gene was *SbEIF4a*, and each reaction was performed in triplicate. The relative expression of *SbNF-YA*s was calculated using the 2^−ΔΔCt^ method (Livak and Schmittgen, 2001), and the gene expression map was drawn using Excel 2019. Primer Premier 6.0 was used to design gene-specific primers, verified by the Oligo 7.0 software and synthesized by Bioengineering (Shanghai) Co., Ltd. The primer sequences for *SbEIF4a* and *SbNF-YA*s are listed in **Supplementary Table S1**.

### *SbNF-YA6* cloning, vector construction, and transformation into *Arabidopsis*

Total RNA was extracted from leaves using an EASY spin Plus Plant RNA Kit (Aidlab Biotech, Beijing, China), and first-strand cDNA was synthesized using MonScript™ RTIII All-in-One Mix with dsDNase (Monad, Shenyang, China). The *SbNF-YA6* gene-specific primers R1f and R1r (**Supplementary Table S1**) were designed using the Primer Premier 6.0 software, and the full-length *SbNF-YA6* coding sequence was amplified using MonAmp™ 2× MonHI-FI HS Mix (Monad, Shenyang, China). The amplification procedure was as follows: 98°C pre-denaturation for 40 s, then 35 cycles of 98°C denaturation for 10 s, 57°C annealing for 30 s, 72°C extension for 30 s, and 72°C final extension for 5 min. After electrophoresis on an agarose gel and purification from the gel slice using a SanPrep Column PCR Product Purification Kit (Sangon, Shanghai, China) kit, the PCR amplicon was ligated into the pUCm-T vector (Sangon, Shanghai, China) and transformed into *Escherichia coli* DH5α (Sangon, Shanghai, China). Plasmid DNA was extracted from PCR-positive colonies using an EasyPure^®^ Plasmid MiniPrep Kit (TRAN, Beijing, China) kit and subjected to Sanger sequencing using primers R2f and R2r (**Supplementary Table S1**). The vector pCAMBIA2301 (Sangon, Shanghai, China) was linearized by digestion with *Kpn*I and mixed with a sequenced-verified pUCm-T-*SbNF-YA6* construct for ligation via homologous recombination using a ClonExpress II One Step Cloning Kit (Vazyme, Jiangsu, China).

The resulting construct was verified by Sanger sequencing with primers R3f and R3r (**Supplementary Table S1**) and transformed into *Agrobacterium* (*Agrobacterium tumefaciens*) strain GV3101 (Sangon, Shanghai, China). After confirmation by colony PCR, YEB medium containing 100 mg/mL kanamycin was inoculated with a positive colony and cultured at 28°C to an OD600 of 0.8–1.5. After centrifugation, the bacterial pellet was resuspended in an osmotic buffer [40 g sucrose, 8 μL 6-benzylaminopurine [6-BA], 2.4 g Murashige and Skoog (MS) salts, and 70 μL Silwet L-77]. The inflorescences of *Arabidopsis* plants were immersed in the bacterial suspension for 2 min using the floral dip method. After incubation in a humid environment in the dark overnight, the transformed plants were moved to a greenhouse for standard culture with periodically repeated infection. The T1 seeds were collected and plated onto half-strength MS medium containing 100 mg/mL kanamycin; resistant seedlings were transplanted into soil. Genomic DNA was extracted from each seedling and genotyped to confirm the presence of the transgene. The seeds for T1 generation plants were harvested to obtain T2 generation transgenic seeds. For each T2 line, 100 seeds were plated onto MS medium containing 100 mg/mL kanamycin to determine the segregation ratio of the transgene; lines with a 3:1 segregation ratio for resistant:sensitive seedlings were selected and their resistant seedlings transplanted to soil. T2 generation plants were self-pollinated, and their T3 generation seeds were collected. The T3 lines *SbNF-YA6*-OE1, *SbNF-YA6*-OE12, and *SbNF-YA6*-OE15 were chosen for experiments.

### Subcellular localization of SbNF-YA6

The full-length *SbNF-YA6* coding sequence without the stop codon was amplified and ligated into the pRI101-GFP vector carrying the sequence for the green fluorescent protein (*GFP*) to obtain the *35S:SbNF-YA6-GFP* construct. The resulting construct and the *35S:GFP* vector (used as control) were individually transformed into *Agrobacterium* strain GV3101. Positive colonies were identified by colony PCR, and plasmid DNA was extracted from bacterial cultures for Sanger sequencing. A plant protoplast isolation kit (Beyotime, Beijing, China) was used to isolate protoplasts from Arabidopsis plants at the five- to six-leaf stage. The *35S:GFP* (control) and *35S:SbNF-YA6-GFP* constructs were individually transfected into protoplasts using a plant protoplast transfection kit (Beyotime, Beijing, China). The subcellular localization of SbNF-YA6 was observed under an FV1000 (Olympus, Hamburg, Germany) confocal microscope.

### Drought tolerance analysis of transgenic Arabidopsis expressing *SbNF-YA6* at the germination stage

Seeds for the wild-type Col and *SbNF-YA6*-OE transgenic Arabidopsis lines (OE1, OE12, and OE15) were placed in a 4°C incubator for stratification for 3 days, disinfected with 75% (v/v) ethanol for 5 min, washed with distilled water three times, and then sown onto half-strength MS medium alone or containing mannitol (100, 200, and 300 mM). All plates were placed in a growth chamber for germination and growth. The temperature was set to 25°C, under a 16-h light/8-h dark photoperiod, and relative humidity of 60%. After 7 days, the germination rate of seeds on each plate was recorded, and the Arabidopsis seedlings were moved to a square Petri plate containing MS medium. After an additional 3 days of growth, root length and fresh weight were measured, and each experiment was performed three times.

### Analysis of drought tolerance of transgenic Arabidopsis expressing *SbNF-YA6* at the seedling stage

Seeds for the wild-type Col and *SbNF-YA6*-OE transgenic Arabidopsis lines (OE1, OE12, and OE15) were sown on half-strength MS medium and cultured in a growth chamber under the same conditions as above. When they had reached the four-leaf stage, seedlings with consistent growth were transferred to half-strength MS medium alone or half-strength MS medium containing 300 mM mannitol. After 2 days of culture, physiological indicators such as O_2_^−^, H_2_O_2_, malondialdehyde (MDA), soluble protein, and proline contents, as well as peroxidase (POD), superoxide dismutase (SOD), and catalase (CAT) activities, were measured. Each experiment was performed three times.

### Analysis of drought tolerance of transgenic Arabidopsis expressing *SbNF-YA6* at the adult stage

Seeds for the wild-type Col and *SbNF-YA6*-OE transgenic Arabidopsis lines (OE1, OE12, and OE15) were sown in the soil. After 1 month of growth under water-replete conditions, watering was withheld to impose drought stress. After 7 days of drought stress, the phenotype of the plants was observed, and photographs were taken. Then, irrigation was resumed. After 7 days, the phenotype of all plants was observed, and photographs were taken.

### RT-qPCR analysis of drought tolerance functional genes in transgenic Arabidopsis expressing *SbNF-YA6*

Seeds for the wild-type Col and *SbNF-YA6*-OE transgenic Arabidopsis lines (OE1, OE12, and OE15) were sown on half-strength MS medium and cultured in a growth chamber. When the seedlings had reached the four-leaf stage, Arabidopsis seedlings with consistent growth were transferred to half-strength MS medium alone or half-strength MS medium containing 300 mM mannitol. After 1 day of culture, samples were collected, frozen in liquid nitrogen, and then stored at −80°C for later use. The reference gene for RT-qPCR analysis was *ACTIN8*, and all reactions were performed in triplicate. All other steps for RT-qPCR analysis were as described above. The primer sequences are listed in **Supplementary Table S1**.

## Results

### Identification and physicochemical property analysis of NF-YA proteins in sorghum

With the help of local Hmmer and BLASTP searches for sorghum proteins harboring a typical CBFB_NFYA Pfam domain, we identified SbNF-YA members, which we designated SbNF-YA1 to SbNF-YA9 (**Table 1**). An analysis of the physicochemical properties of the predicted SbNF-YA proteins indicated that SbNF-YA9 is the largest, with 419 amino acid (aa) residues and a molecular mass of 45.47 kDa, and SbNF-YA8 is the smallest, with 213 aa residues and a molecular mass of 23.17 kDa. The isoelectric points of SbNF-YAs range from 7.97 to 10.99, classifying them as alkaline proteins (pI > 7.5). The fat coefficients are 49.11–68.71, the instability coefficients are 56.48–72.53, and the hydrophilicity index is from −1.087 to −0.369. Notably, all nine SbNF-YA members exhibited an instability coefficient above 40 and a hydrophilicity index below 0, suggesting that they are unstable and hydrophilic. Subcellular localization predictions suggested that, with the exception of SbNF-YA9 (predicted to localize inside and outside chloroplasts), all other SbNF-YAs are likely nuclear proteins. Secondary structure predictions revealed the presence of α-helices, extended chains, β-sheets, and random coils in all SbNF-YAs (**Table 2**). The α-helices and random coils were the predominant structures in each protein, with β-sheets and extended chain structures being comparatively scarce. Specifically, random coils and α-helices comprised 46.3%–64.45% and 17.4%–33.8% of each protein structure, respectively, while extended chains accounted for 9.0%–18.75%, and β-sheets 3.65%–6.6%.

**Table 1:** Gene information and subcellular localization of NF-YA family in sorghum.

Gene name	Sequence ID	Number of amino acids/aa	Molecular weight/ku	pI	Instability index	Aliphatic index	Grand average of hydropathicity	Subcellular localization
*SbNF-YA1*	Sobic.001G127800.1	267	28.09	9.57	59.18	63.30	−0.710	Nucleus
*SbNF-YA2*	Sobic.001G154500.1	345	36.52	9.43	59.55	57.51	−0.524	Nucleus
*SbNF-YA3*	Sobic.001G340200.1	309	33.89	9.65	61.60	60.94	−0.723	Nucleus
*SbNF-YA4*	Sobic.001G486000.1	248	27.00	8.03	67.78	53.99	−0.840	Nucleus
*SbNF-YA5*	Sobic.002G038500.1	287	30.53	9.74	57.78	60.84	−0.537	Nucleus
*SbNF-YA6*	Sobic.002G370800.1	301	32.78	8.50	58.91	56.41	−0.814	Nucleus
*SbNF-YA7*	Sobic.004G316500.2	304	33.13	10.99	56.48	53.39	−0.751	Nucleus
*SbNF-YA8*	Sobic.008G168300.1	213	23.17	7.97	72.53	49.11	−1.087	Nucleus
*SbNF-YA9*	Sobic.008G174650.1	419	45.47	9.38	58.26	68.71	−0.369	Chloroplast

**Table 2:** The secondary structure of sorghum NF-YA family proteins.

Protein	Alpha helix/%	Extended strand/%	Beta turn/%	Random coil/%
SbNF-YA1	22.10	10.11	3.75	64.04
SbNF-YA2	19.13	12.75	5.51	62.61
SbNF-YA3	21.68	10.36	6.15	61.81
SbNF-YA4	20.97	12.90	5.24	60.89
SbNF-YA5	33.80	13.24	6.62	46.34
SbNF-YA6	22.92	8.97	3.65	64.45
SbNF-YA7	19.41	18.75	6.25	55.59
SbNF-YA8	20.66	9.39	6.10	63.85
SbNF-YA9	17.42	15.75	3.82	63.01

### Analysis of gene structure and conserved domain of the SbNF-YA family

**Figure 1:**
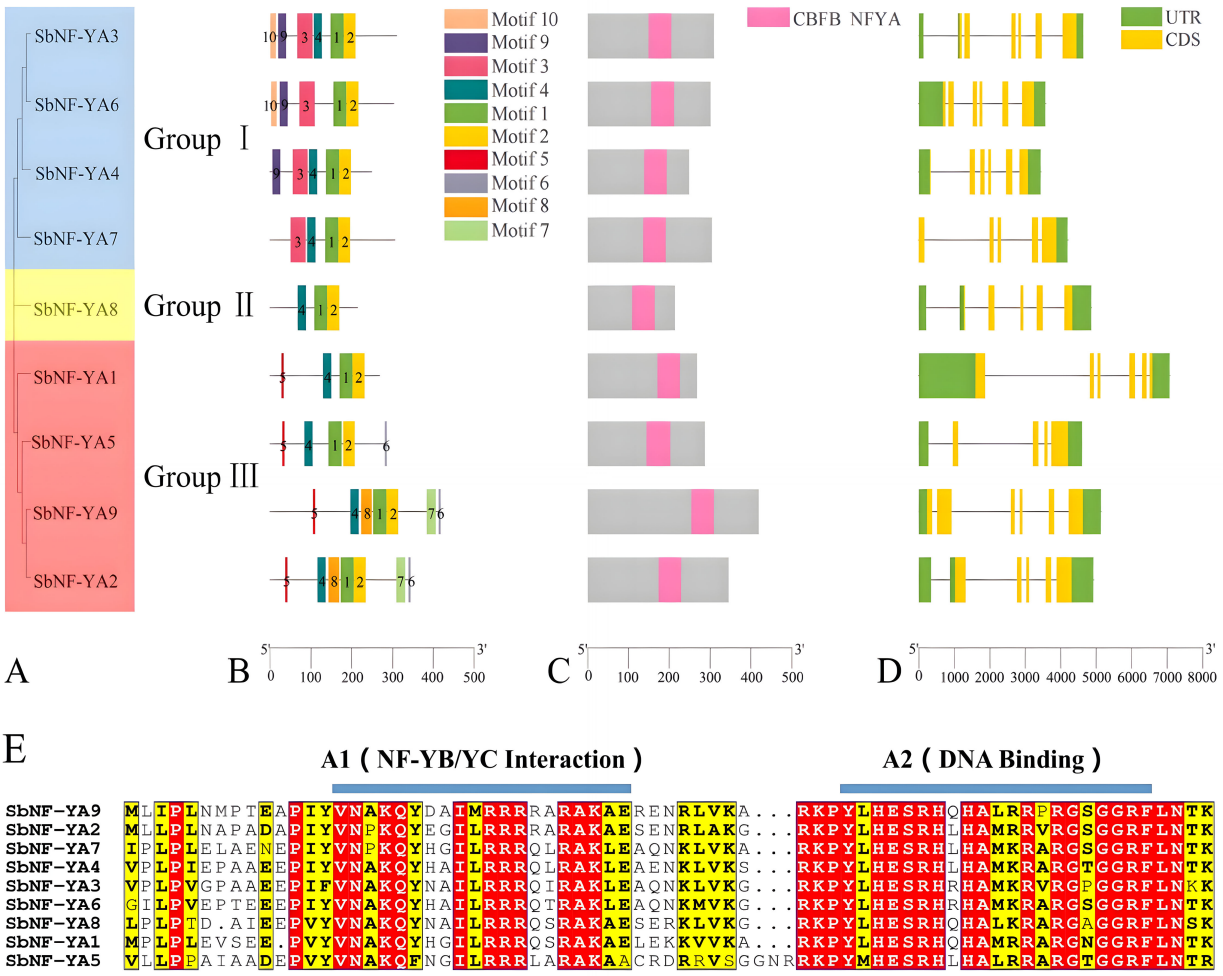
Characterization of the *NF-YA* gene and NF-YA protein family in sorghum. **(A)** Unrooted phylogenetic tree. **(B)** MEME suite analysis of the conserved motifs present in SbNF-YAs. **(C)** Diagram of SbNF-YAs showing the position of the CBFB_NFYA domain. **(D)** Gene structure of *SbNF-YA*s. **(E)** Amino acid sequence alignment of the core domain of SbNF-YA members.

To elucidate the evolutionary relationship among the nine SbNF-YA family proteins, we reconstructed a phylogenetic tree using their full-length amino acid sequences (**Figure 1A**). The nine SbNF-YAs cluster into three distinct groups: Group I comprised SbNF-YA3, SbNF-YA4, SbNF-YA6, and SbNF-YA7; Group II was represented by SbNF-YA8; and Group III consisted of SbNF-YA1, SbNF-YA2, SbNF-YA5, and SbNF-YA9 (**Figure 1A**). Furthermore, each *SbNF-YA* member possessed a unique intron–exon configuration. Nevertheless, members within each subgroup showed similar exon–intron structures, whereas those from distinct subgroups displayed more varied exon–intron composition patterns (**Figure 1D**). Genes from Group I, with the exception of *SbNF-YA7*, as well as *SbNF-YA8* from Group II, each have five exons; *SbNF-YA7* has six exons. Genes from Group III, except *SbNF-YA5*, have four exons, while *SbNF-YA5* has five to six exons.

We looked for conserved functional domains in SbNF-YA using the MEME suite, which showed that members within the same subgroup also share similar conserved motifs (**Figure 1B**). Notably, motifs 3, 9, and 10 are exclusive to Group I members, whereas motifs 5, 6, 7, and 8 are unique to Group III. These distinct motifs may contribute to unique functions among SbNF-YA members. Meanwhile, motifs 1 and 2 are present in all SbNF-YA members and together form the CBFB_NFYA core conserved domain that is characteristic of NF-YAs, although it occupies different positions in each SbNF-YA member (**Figure 1C**). The CBFB_NFYA domain comprises 53 aa and is characterized by two highly conserved regions, denoted as A1 and A2, along with an intervening 12-aa linker region (**Figure 1E**). The A1 domain is an α-helix of 20 aa, positioned at the N-terminus of the core region, and serves as the interaction interface to which NF-YB and NF-YC bind. The A2 domain is another α-helix of 21 aa, which is the DNA-binding domain crucial for the specific recognition of the CCAAT box within the promoters of target genes.

### Phylogenetic analysis of NF-YA members from sorghum, *Arabidopsis*, rice, maize, soybean, and foxtail millet

**Figure 2:**
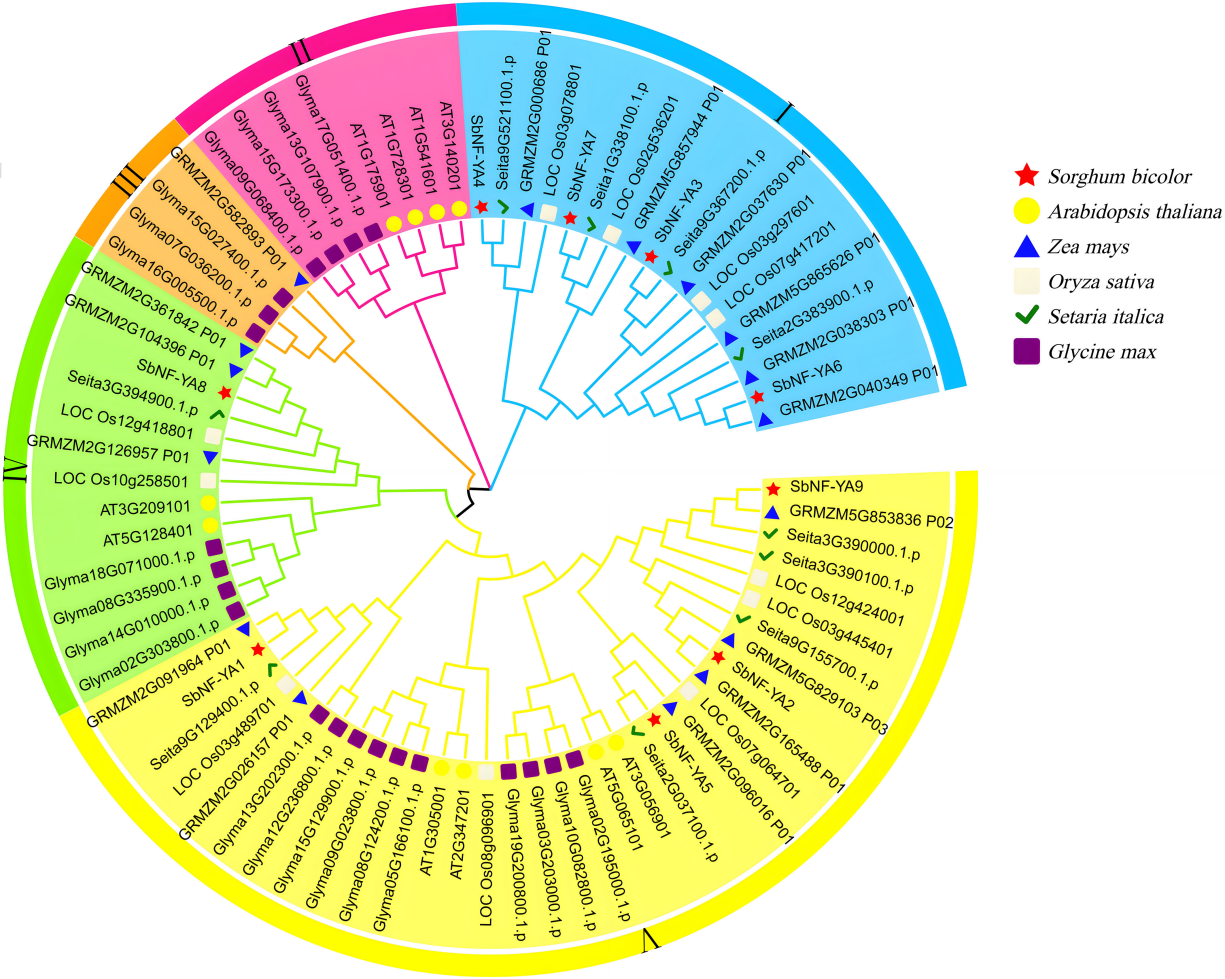
Phylogenetic tree of NF-YA proteins in sorghum (*Sorghum bicolor*), Arabidopsis (*Arabidopsis thaliana*), rice (*Oryza sativa*), maize (*Zea mays*), millet (*Setaria italica*), and soybean (*Glycine max*). The NF-YA members from each plant species are indicated by different symbols; colors indicate species.

To explore the evolutionary relationship between SbNF-YA and NF-YAs from other plant species, we reconstructed a phylogenetic tree using NF-YAs from sorghum, Arabidopsis, rice, maize, soybean, and foxtail millet to infer the origin and evolution of SbNF-YA members (**Figure 2**). To this end, we obtained the amino acid sequences of 77 NF-YAs from all six species, which clustered into five distinct groups. Cluster V had the most NF-YA members, with four SbNF-YAs, four AtNF-YAs, six ZmNF-YA, five OsNF-YAs, five SiNF-YAs, and 10 GmNF-YAs. By contrast, Cluster III had the fewest members, with only three GmNF-YAs and one ZmNF-YA, and Cluster II comprised solely four AtNF-YAs and four GmNF-YAs; neither cluster had members in sorghum. Clusters I and IV included four and one SbNF-YAs, respectively. Thus, SbNF-YAs were distributed across all clusters except Clusters II and III. Moreover, several SbNF-YAs are grouped with NF-YAs from maize and foxtail millet, while no SbNF-YA is present in the clusters containing Arabidopsis and soybean NF-YAs, consistent with the closer genetic relationship of sorghum to maize and foxtail millet compared to Arabidopsis and soybean.

### Analysis of the chromosomal distribution of *SbNF-YA* genes, duplications, and colinearity between species

**Figure 3:**
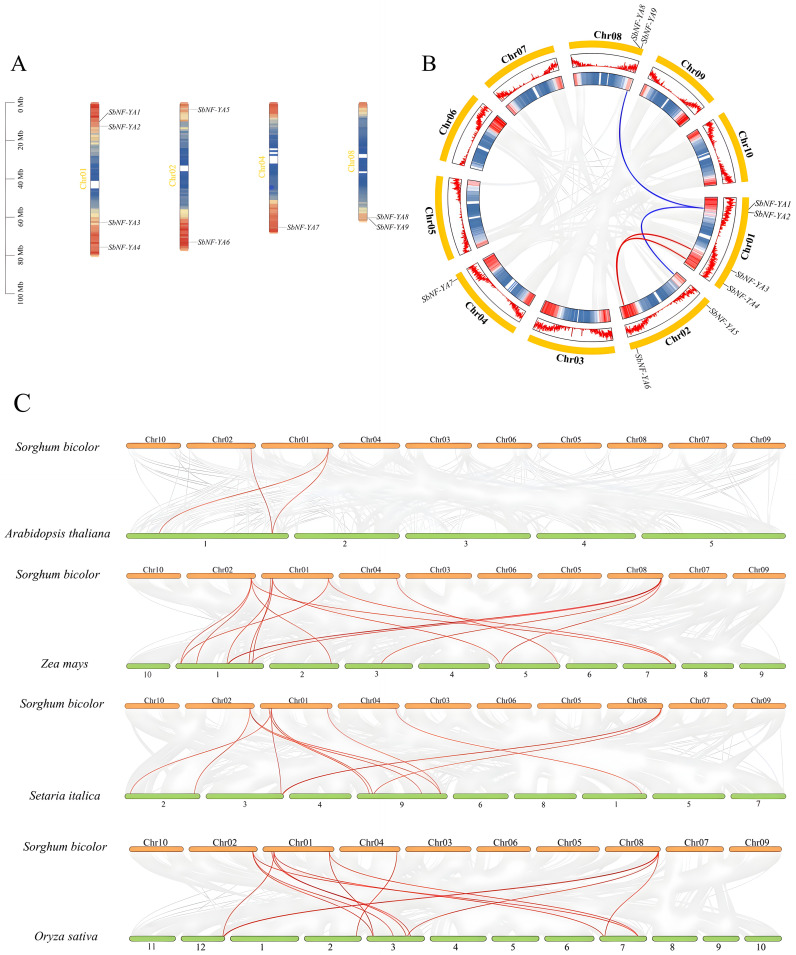
Chromosomal distribution of *SbNF-YA* genes and their colinearity relationships with individual *SbNF-YA* genes and with *NF-YA* genes from other plant species. **(A)** Position of *NF-YA* genes along the chromosomes of the sorghum genome. **(B)** Colinearity relationship among *SbNF-YA* genes. Red lines, The expression genes *SbNF-YA3*, *SbNF-YA4* and *SbNF-YA6* have a collinear relationship. Blue lines, The expression genes *SbNF-YA2*, *SbNF-YA5* and *SbNF-YA9* have a collinear relationship. **(C)** Colinearity relationship between *SbNF-YA* genes and *NF-YA* genes from Arabidopsis (*Arabidopsis thaliana*), maize (*Zea mays*), millet (*Setaria italica*), and rice (*Oryza sativa*).

The nine *SbNF-YA* genes are unevenly distributed across four of the 10 chromosomes of the sorghum genome (**Figure 3A**). Specifically, chromosome 1 harbors the highest number of *SbNF-YA* genes, with four members, followed by chromosomes 2 and 8, with two genes each; chromosome 4 carries one *SbNF-YA* gene. Overall, the *SbNF-YA* genes are predominantly located near chromosome ends, with *SbNF-YA1*, *SbNF-YA4*, *SbNF-YA6*, and *SbNF-YA7* being situated within regions with high gene density. To decipher the amplification mechanism of the *SbNF-YA* family, we used MCScanX to examine whether and which *SbNF-YA* genes arose from tandem duplication or fragment duplication (**Figure 3B**). We detected no evidence of tandem duplication events among these genes, but we identified dispersed duplication events. Specifically, the following gene pairs arose from dispersed duplication events: *SbNF-YA1* on chromosome 1 and *SbNF-YA9* on chromosome 8; *SbNF-YA2* on chromosome 1 and *SbNF-YA5* on chromosome 2; *SbNF-YA3* and *SbNF-YA4* on chromosome 1 and *SbNF-YA6* on chromosome 2. We conclude that gene duplication events may have played an important role in the amplification and evolution of the *SbNF-YA* gene family, whose members may therefore have certain similarities in structure and function.

To further analyze the similarities and evolutionary relationship of *SbNF-YA* genes, we explored the colinearity between *SbNF-YA* genes in sorghum and their putative orthologs in the genomes of Arabidopsis, maize, foxtail millet, and rice (**Figure 3C**). We detected colinearity relationships between two *SbNF-YA* genes and two Arabidopsis *NF-YA* genes, seven *SbNF-YA* genes and 13 maize *NF-YA* genes, seven *SbNF-YA* genes and 10 foxtail millet *NF-YA* genes, and seven *SbNF-YA* genes and 11 rice *NF-YA* genes. As suggested by the phylogenetic analysis (**Figure 2**), the greater colinearity between *NF-YA* genes from sorghum and maize is consistent with the closer genetic relationship between sorghum and maize and the more distant genetic relationship between sorghum and Arabidopsis.

### Analysis of *cis*-acting elements in the *SbNF-YA* promoters

**Figure 4:**
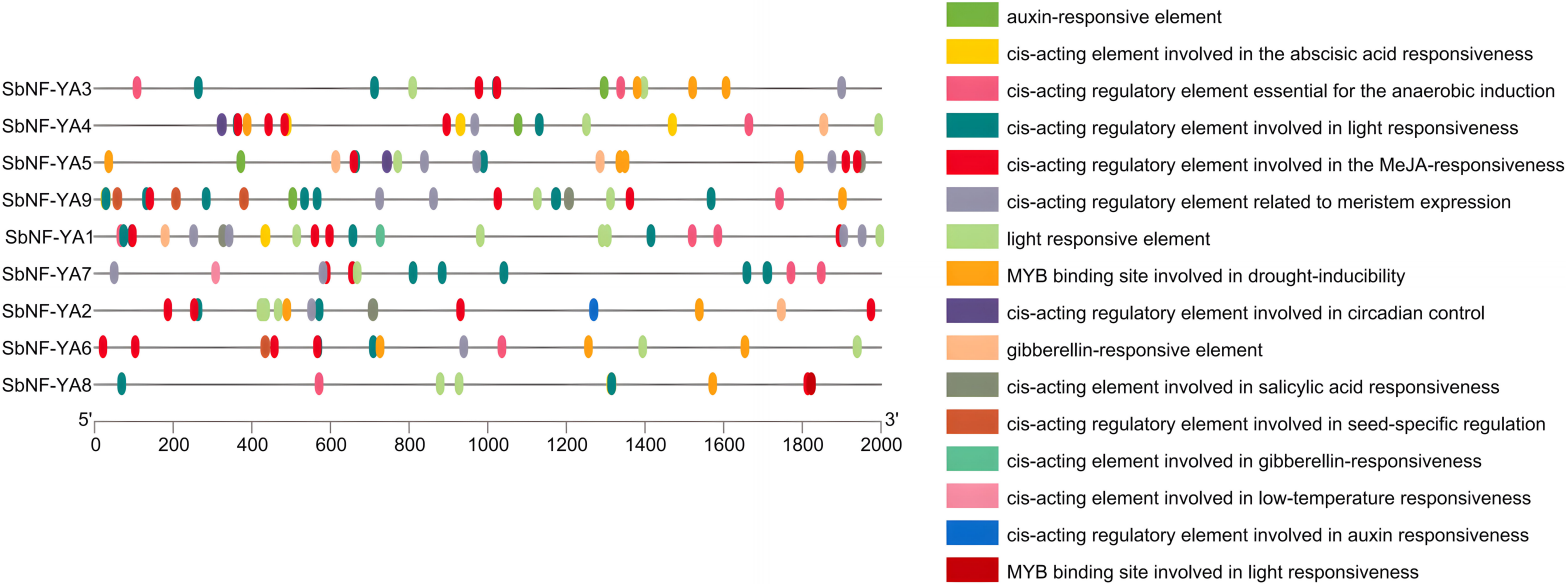
Predicted *cis*-acting elements in the promoters of *SbNF-YA* genes. A 2,000-bp promoter fragment upstream of the transcription start site was analyzed for the presence of *cis*-regulatory elements via the PlantCare database (http://bioinformatics.psb.ugent.be/webtools/plantcare/html/).

*cis*-Acting regulatory elements are bound by specific transcription factors, thereby regulating transcription and modulating plant responses to stress, among other processes (Wittkopp and Kalay, 2012). To obtain a glimpse into the possible biological functions of *SbNF-YA* family genes, we looked for *cis*-elements in a 2,000-bp promoter region upstream of the transcription start site of each *SbNF-YA* gene. An analysis by the PlantCare database showed that the promoters of *SbNF-YA* genes contain several phytohormone-related regulatory elements, stress response elements, and light response elements (**Figure 4**). The promoters of all *SbNF-YA* genes contain *cis*-acting regulatory elements that are responsive to the phytohormones ABA and methyl jasmonate (MeJA) and to light, suggesting that the *SbNF-YA* family is involved in these signaling pathways. Additionally, the promoters of four *SbNF-YA* genes appear to possess *cis*-acting elements that are responsive to the other phytohormones, auxin, gibberellin, and salicylic acid, indicating potential roles in mediating phytohormone signaling. Furthermore, the promoters of 2000 bp upstream of the initiation site *SbNF-YA* genes contain putative *cis*-elements associated with response to drought, low temperature, and anaerobic stress, suggesting the involvement of *SbNF-YA* genes in stress response mechanisms. Moreover, we identified *cis*-acting elements related to growth and development, seed-specific regulation, and circadian rhythms in *SbNF-YA* promoters, suggesting that *SbNF-YA* genes may play roles in plant growth and development, phytohormone regulation, and stress responses.

### MiRNA targeting of *SbNF-YA* transcripts by miRNAs and protein interaction network analysis of SbNF-YA proteins

**Figure 5:**
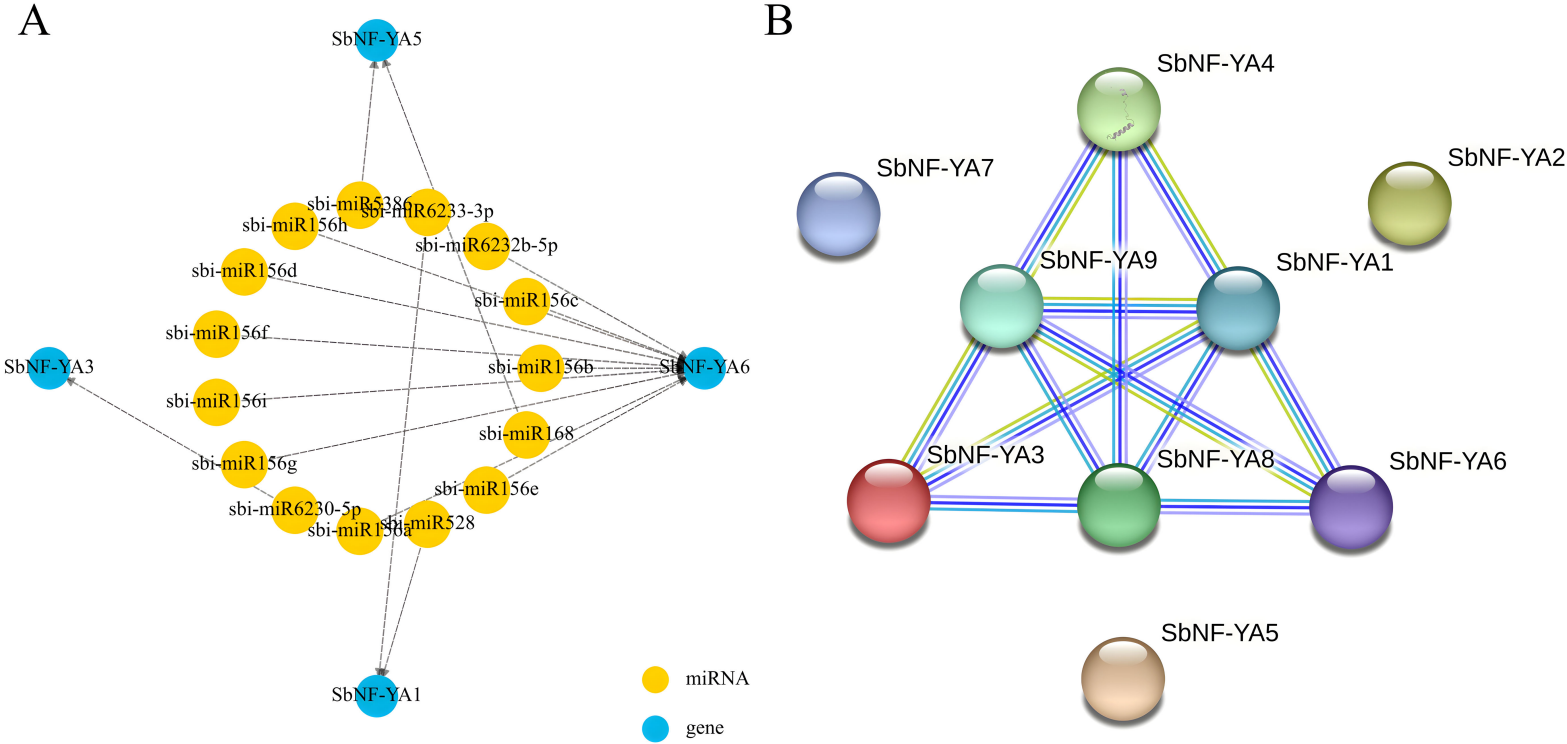
Predictions of *SbNF-YA*–miRNA interactions and protein–protein interactions among SbNF-YAs. **(A)** Diagram showing the predicted *SbNF-YA* transcript–miRNA network. **(B)** Putative protein–protein interaction network among SbNF-YAs as predicted by the STRING database.

MiRNAs are critical regulators of gene expression, with each miRNA potentially modulating the expression (broadly defined) of multiple target genes; a single gene may also be regulated by multiple miRNAs. In this study, we investigated the potential miRNAs targeting the transcripts of individual *SbNF-YA* genes based on analysis via the psRNATtarget database. We thus identified miRNAs with sequence complementarity to four out of the nine *SbNF-YA* genes (**Figure 5A**). Notably, the transcripts of *SbNF-YA6* may be targeted by multiple miRNAs, namely, sbi-miR156e, sbi-miR156a, sbi-miR156b, sbi-miR156c, sbi-miR156f, sbi-miR156g, sbi-miR156h, sbi-miR156i, sbi-miR156d, and sbi-miR6232b-5p, suggesting that SbNF-YA6 may possess diverse functional roles. The transcripts of *SbNF-YA1* may be targeted by sbi-miR6233-3p and sbi-miR528, while the transcripts of *SbNF-YA5* show sequence complementarity to sbi-miR168 and sbi-miR5386. Finally, the transcripts of *SbNF-YA3* may be targeted by sbi-miR6230-5p. Predicting the miRNAs potentially binding to *SbNF-YA* transcripts for post-transcriptional regulation will be helpful in understanding the role of each *SbNF-YA* gene in plant growth and development and stress tolerance.

Proteins typically engage in protein–protein interactions (PPIs) that regulate various biological activities and functions (Zhao et al., 2020). To obtain more clues about the possible function of SbNF-YAs, we queried the STRING database for the interaction network of the nine SbNF-YA members. Using a medium confidence level of 0.400, the STRING database reported interactions between six of the nine SbNF-YAs (**Figure 5B**). SbNF-YA2, SbNF-YA5, and SbNF-YA7 did not interact with each other or with the other SbNF-YAs, while SbNF-YA1, SbNF-YA8, and SbNF-YA9 were engaged in interactions with five other SbNF-YAs each, indicating that they have similar functions.

### Tissue-specific expression of *SbNF-YA* genes

**Figure 6:**
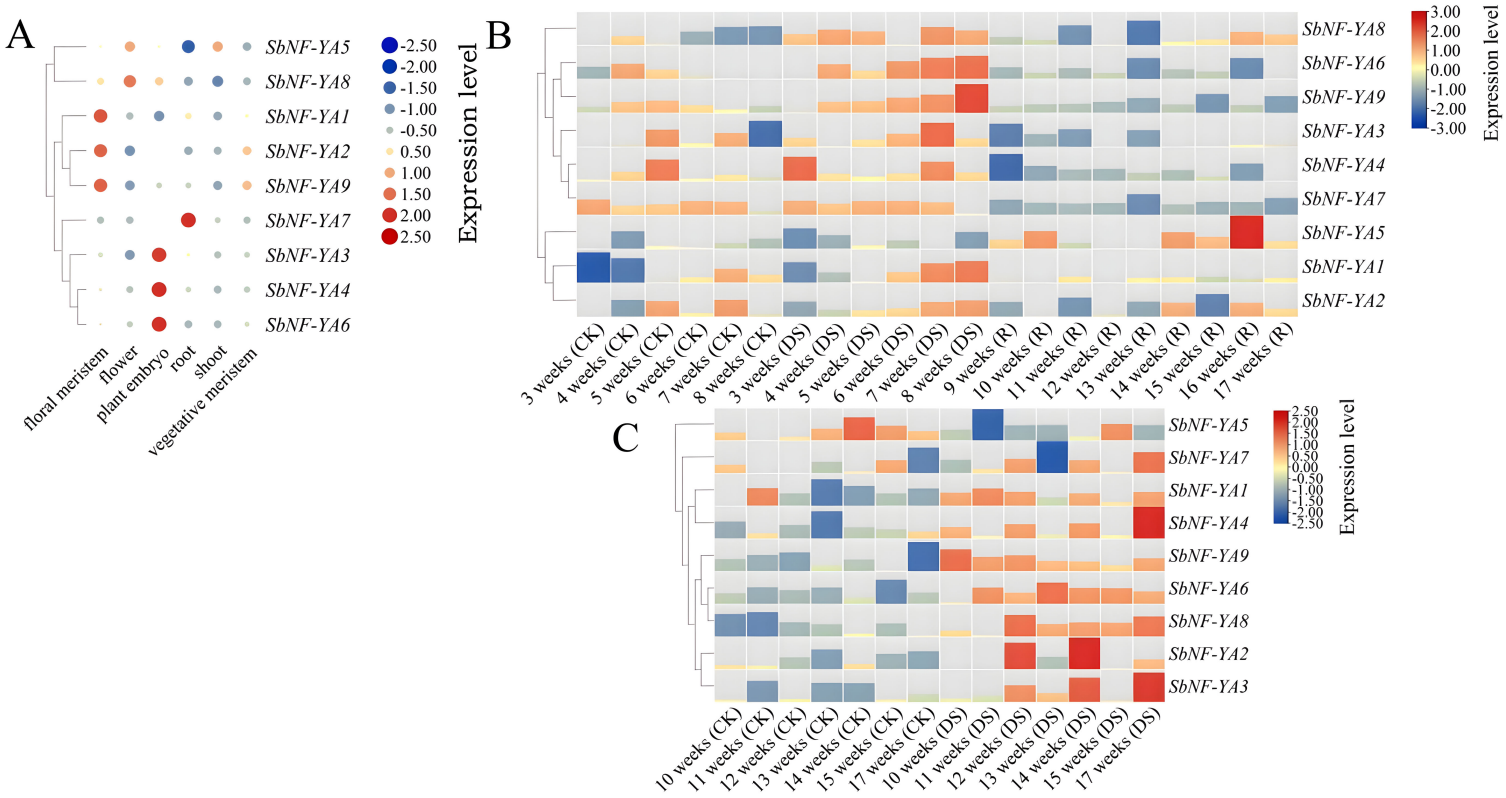
Expression analysis of *SbNF-YA* genes. **(A)** Expression of *SbNF-YA* genes in different tissues of the sorghum variety (BTx623). **(B, C)** Expression of *SbNF-YA* genes in sorghum plants exposed to drought stress before flowering **(B)** or after flowering **(C)**. CK, normal irrigation; DS, drought stress; R, rehydration treatment. In all panels, expression levels were normalized to -3 to 3.

Tissue-specific transcriptome data can be used to examine the expression patterns of specific genes in certain growth and development activities (Islam et al., 2022). We downloaded transcriptome deep sequencing (RNA-seq) data for various sorghum tissues and extracted the expression patterns of all *SbNF-YA* genes (**Figure 6A**). All nine *SbNF-YA* genes were expressed in various tissues, albeit at varying levels. Notably, *SbNF-YA7* exhibited elevated expression in roots, suggesting a potential role in plant stress tolerance through the modulation of the expression of stress-related genes in roots under adverse conditions. *SbNF-YA8* was highly expressed in embryos, flowers, and floral meristems, while *SbNF-YA5* was predominantly expressed in flowers and shoots. Additionally, *SbNF-YA3*, *SbNF-YA4*, and *SbNF-YA6* demonstrated high expression in embryos. *SbNF-YA1* was highly expressed in floral meristems and to a lesser extent in roots, whereas *SbNF-YA2* and *SbNF-YA9* were predominantly expressed in floral and vegetative meristems. In conclusion, the *SbNF-YA* genes are expressed to various levels in embryos, floral meristems, and vegetative meristems, suggesting that they may facilitate plant growth under adverse conditions.

### Expression of *SbNF-YA* genes under drought stress

To investigate the expression pattern of *SbNF-YA* genes under drought stress conditions, we downloaded published RNA-seq data from two sorghum varieties grown under normal irrigation or subjected to drought stress before or after flowering. We then extracted the expression levels of *SbNF-YA* genes and visualized the data as a heatmap of the log_2_(fold change) values between drought conditions and normal conditions (**Figures 6B, C**). The *SbNF-YA* genes exhibited increased expression levels during drought stress treatment imposed before and after flowering, compared to normal irrigation conditions. Notably, following drought stress prior to flowering followed by rewatering, the expression levels of *SbNF-YA* genes were substantially lower during rewatering than during the drought stress period, suggesting a specific role for *SbNF-YA* genes in the drought stress response. During the preflowering drought stress treatment, the expression of *SbNF-YA1*, *SbNF-YA2*, *SbNF-YA3*, *SbNF-YA6*, and *SbNF-YA9* was markedly upregulated, while that of *SbNF-YA4*, *SbNF-YA7*, and *SbNF-YA8* was moderately upregulated. The expression level of *SbNF-YA5* remained consistently low during the drought stress treatment but rose during rewatering, indicating that *SbNF-YA5* expression may be inhibited under drought stress conditions. The other *SbNF-YA* genes were upregulated during drought stress treatment after flowering, except for *SbNF-YA5*, which was not significant. In summary, except for *SbNF-YA5*, the different *SbNF-YA* genes appear to function in drought stress.

### RT-qPCR verification of *SbNF-YA* expression under drought stress

**Figure 7:**
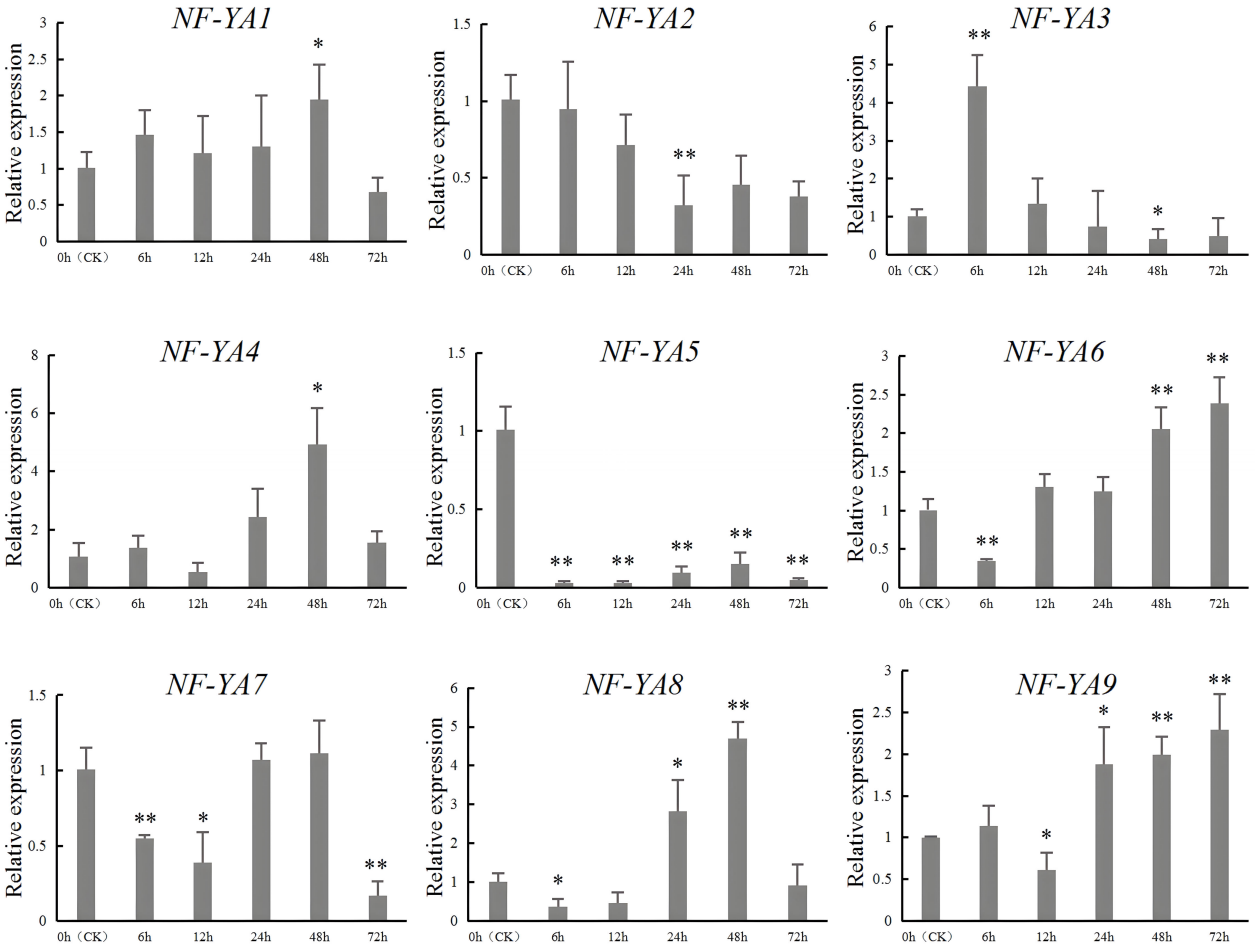
RT-qPCR analysis of *SbNF-YA* genes under drought stress. Leaves from seedlings of the sorghum variety ‘9704X3’ were collected immediately before (0 h, CK) or at 6, 12, 24, 48, and 72 h into simulated drought treatment imposed by 300 mM mannitol. Significant differences (* *p* < 0.05, ** *p* < 0.01) were determined by an independent two-sample *t*-test. Values are means ± standard deviation from 3 technical repetitions replicates.

To verify the RNA-seq results of *SbNF-YA* genes under the drought stress above, we conducted RT-qPCR using seedlings from the drought-tolerant sorghum variety 9704X3 grown in a hydroponic system and subjected to simulated drought stress with PEG-6000 (**Figure 7**). We determined that the expression levels of six of the nine genes (*SbNF-YA1*, *SbNF-YA3*, *SbNF-YA4*, *SbNF-YA6*, *SbNF-YA8*, and *SbNF-YA9*) were upregulated in response to simulated drought stress. Among them, *SbNF-YA1* and *SbNF-YA4* expression levels peaked at 48 h into PEG treatment. The expression levels of *SbNF-YA3* reached a peak at 6 h into PEG treatment and then gradually decreased over the next 66 h. By contrast, *SbNF-YA6* expression gradually rose after 12 h into treatment and reached a peak at 72 h. The expression of *SbNF-YA8* peaked at 48 h into simulated drought stress. *SbNF-YA9* expression gradually and slowly increased from a trough at 12 h into treatment to reach a peak after 72 h into simulated drought stress. Of the remaining genes, *SbNF-YA2* and *SbNF-YA7* expression levels were downregulated, with *SbNF-YA2* expression levels being significantly lower than those in the control at 24 h into treatment. *SbNF-YA7* expression was the lowest at 72 h into stress and also demonstrated significantly lower levels at 6 h and 12 h. These findings suggest that *SbNF-YA2* and *SbNF-YA7* have negative regulatory roles during stress responses. *SbNF-YA5* expression was repressed during simulated drought stress. In summary, consistent with the RNA-seq analysis, the expression of eight of the *SbNF-YA* genes (with *SbNF-YA5* being the exception) responded to drought stress to various degrees, indicating that these genes play a role in the drought response of sorghum.

### Cloning of *SbNF-YA6* and subcellular localization of SbNF-YA6

**Figure 8:**
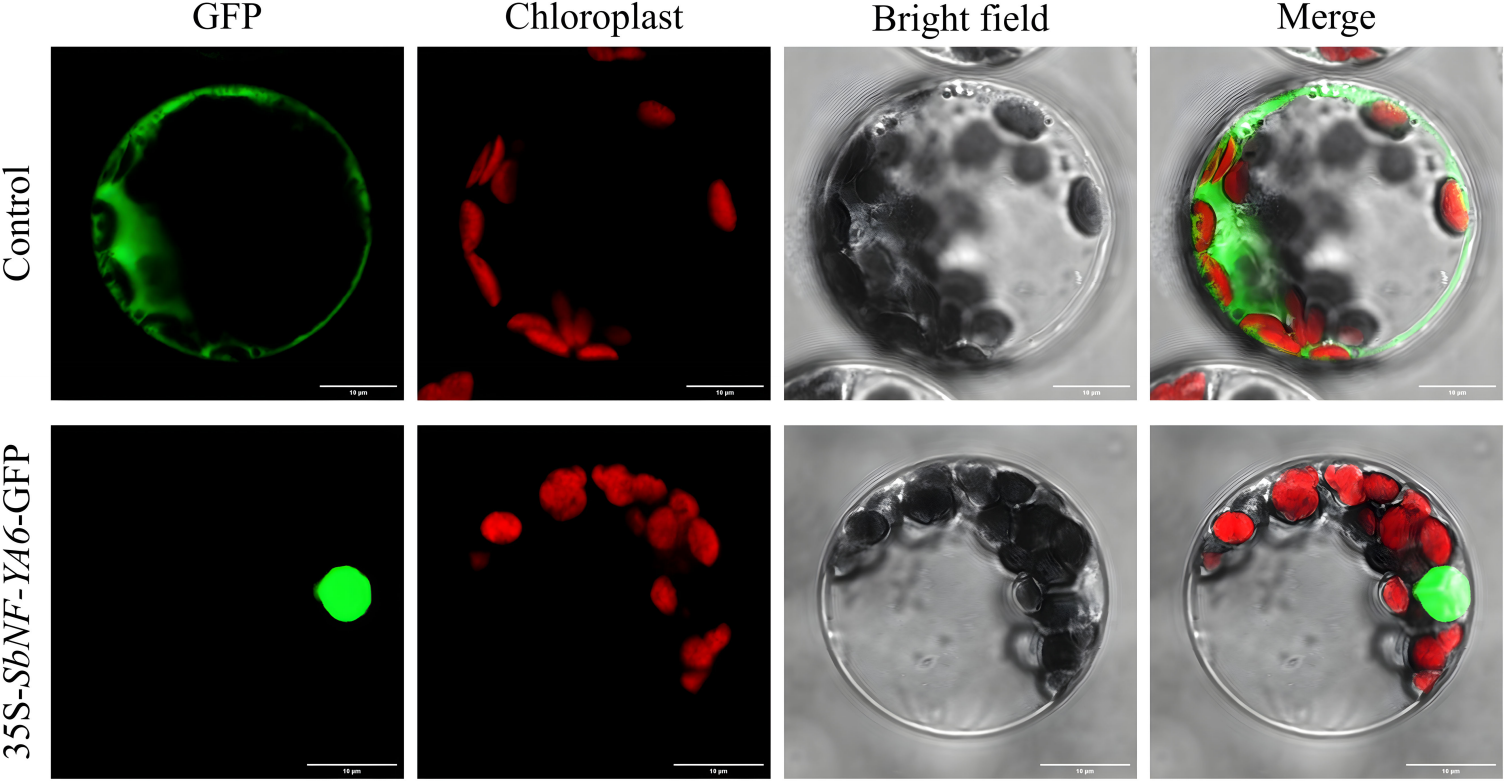
Subcellular localization of SbNF-YA6. Arabidopsis protoplasts were transfected with *35S:GFP* (control) or *35S:SbNF-YA6*-GFP. The fluorescence signals were detected under a confocal microscope. Chlorophyll autofluorescence was used to visualize chloroplasts.

Of the nine *SbNF-YA* genes, *SbNF-YA6* expression was the most highly induced by simulated drought stress, prompting us to focus on this gene for functional characterization. Accordingly, we cloned the *SbNF-YA6* cDNA by RT-PCR (**Supplementary Figure S1**). The *SbNF-YA6* coding sequence is 906 bp in length and encodes a protein of 301 amino acids, which is consistent with the genome sequence and annotation. To assess the subcellular localization of SbNF-YA6, we cloned the full-length *SbNF-YA6* coding sequence without the stop codon in-frame and upstream of the sequence encoding the GFP, resulting in the *35S:SbNF-YA6-GFP* plasmid, which we transfected into Arabidopsis protoplasts. As a control, we also transfected protoplasts with a *35S:GFP* plasmid. The green fluorescence detected for the *35S:GFP* plasmid was uniformly distributed in the cell. By contrast, we observed green fluorescence predominantly in the nucleus of protoplasts transfected with the *35S:SbNF-YA6-GFP* construct (**Figure 8**). This observation suggests that SbNF-YA6 is a nuclear protein.

### Effects of *SbNF-YA6* heterologous expression in Arabidopsis on drought tolerance at the germination stage

**Figure 9:**
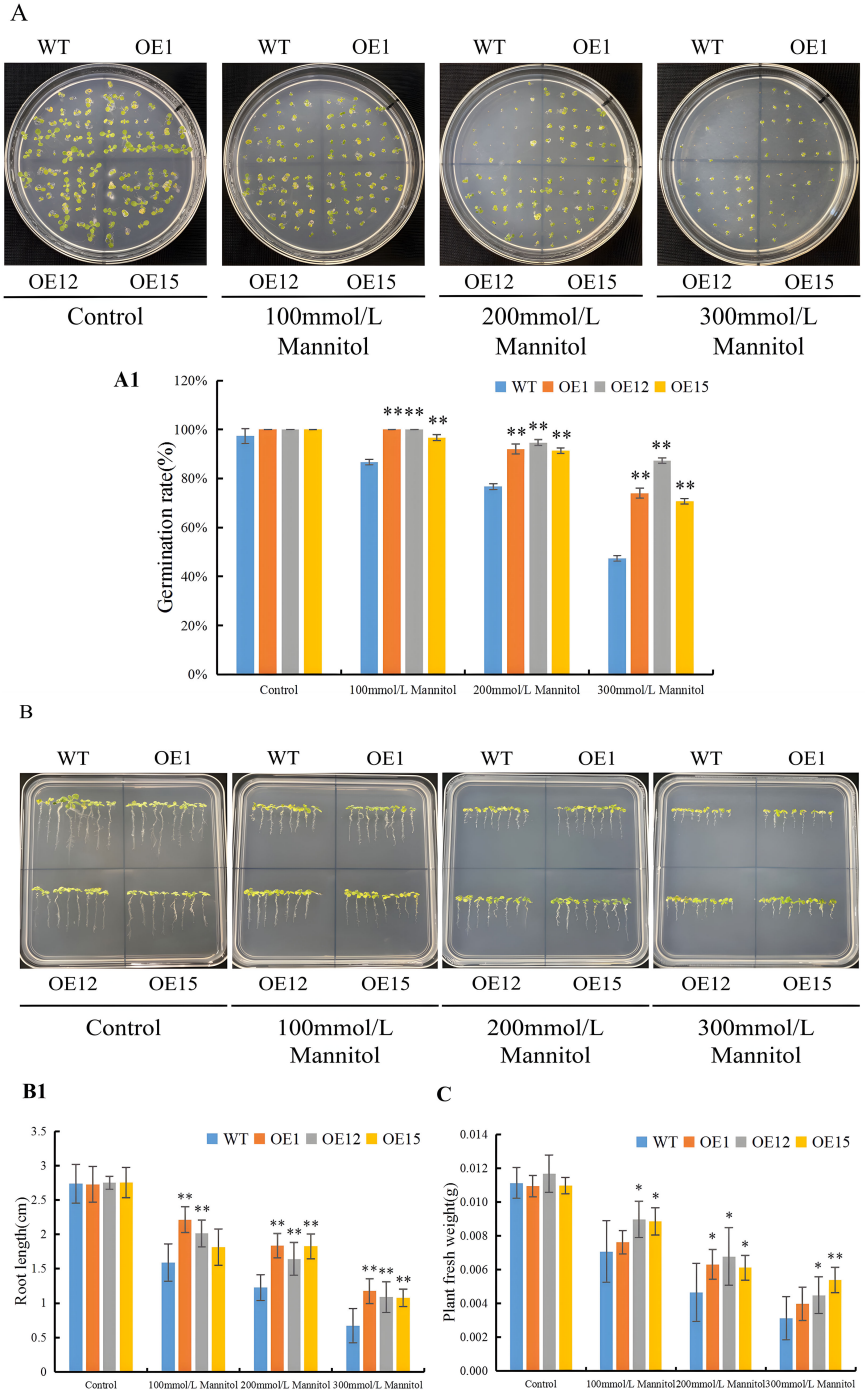
Effect of heterologous *SbNF-YA6* expression in Arabidopsis on drought tolerance at the germination stage. **(A, A1)** Representative photographs **(A)** and mean germination rate **(A1)** of seeds from the wild type Col and transgenic Arabidopsis *SbNF-YA6*-OE lines following germination on MS medium alone (Control) or containing 100, 200, or 300 mM mannitol to simulate drought stress. **(B, B1)** Representative photographs **(B)** and mean root length **(B1)** of seedlings from the wild-type xx and transgenic Arabidopsis *SbNF-YA6*-OE lines under drought stress. **(C)** Mean fresh weight of seedlings from the wild-type Col and transgenic Arabidopsis *SbNF-YA6*-OE lines under simulated drought stress. Values are means ± standard deviation from 3 biological repetitions independent experiments. Significant differences were determined by an independent two-sample *t*-test (* *p* < 0.05, ** *p* < 0.01).

To assess the function of SbNF-YA6 in drought tolerance, we generated transgenic Arabidopsis lines heterologously expressing the gene under the control of the 35S promoter. We chose three lines, OE1, OE12, and OE15, with high expression levels of *SbNF-YA6* and carrying a single transgene. We sowed seeds of the wild-type Col and each of the three transgenic lines on MS medium alone or containing the osmoticum mannitol. We scored the germination rates of all seeds after 7 days of incubation. When germinated on MS medium, the wild type and the three transgenic Arabidopsis lines (OE1, OE12, and OE15) had comparable germination rates. When sown on half-strength MS medium containing 100, 200, or 300 mM mannitol, however, the transgenic seeds germinated at significantly higher rates than the wild-type seeds (**Figures 9A, A1**). As another means to assess the effect of *SbNF-YA6* expression on tolerance to mannitol treatment, we measured the root length of seedlings grown on MS medium alone or containing mannitol. In the absence of mannitol, the root lengths of wild-type and transgenic seedlings were similar. In the presence of mannitol, however, the roots of the transgenic lines were generally significantly longer (**Figures 9B, B1**). We obtained similar results with seedling fresh weight, with no significant differences observed among the transgenic lines and the wild type in the absence of mannitol. However, when grown on half-strength MS medium containing 100, 200, or 300 mM mannitol, the fresh weights of the transgenic lines (OE1, OE12, and OE15) were significantly higher than those of the wild type (**Figure 9C**).

### Effects of *SbNF-YA6* heterologous expression in Arabidopsis on drought tolerance at the seedling and adult stages

**Figure 10:**
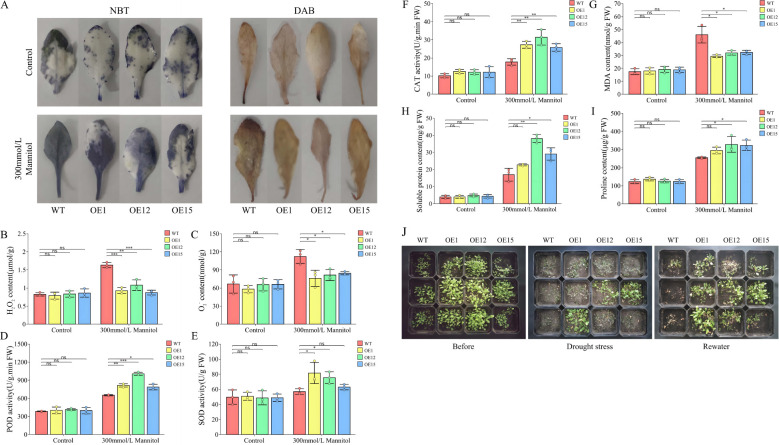
Effects of heterologous *SbNF-YA6* expression in Arabidopsis on seedling phenotypes and physiological indices. **(A)** Representative photographs of leaves from the wild-type Col and transgenic Arabidopsis *SbNF-YA6*-OE lines stained with nitroblue tetrazolium (NBT) or 3,3′-diaminobenzidine (DAB). **(B–I)** H_2_O_2_ content **(B)**, O_2_^−^ content **(C)**, peroxidase (POD) activity **(D)**, superoxide dismutase (SOD) activity **(E)**, catalase (CAT) activity **(F)**, malondialdehyde (MDA) content **(G)**, soluble protein content **(H)**, and proline content **(I)**. Values are means ± standard deviation from 3 biological repetitions independent experiments. Significant differences were determined by an independent two-sample *t*-test (ns, not significant, * *p* < 0.05, ** *p* < 0.01, *** *p* < 0.001). **(J)** Representative photographs of wild-type and Arabidopsis *SbNF-YA6*-OE lines before and after drought stress and after rewatering.

To further elucidate the effects of the heterologous expression of *SbNF-YA6* on drought stress, we stained the leaves of wild-type and transgenic Arabidopsis seedlings grown under normal conditions or drought stress treatment, imposed by withholding irrigation, with nitroblue tetrazolium (NBT) and 3,3′-diaminobenzidine (DAB). The leaves of all genotypes showed similar staining with NBT or DAB, but the leaves of wild-type seedlings were darker than those of the transgenic lines under drought stress treatment (**Figure 10A**), indicating that the transgenic lines accumulate lower levels of H_2_O_2_ and O_2_^−^, suggestive of improved reactive oxygen species (ROS) scavenging ability. We focused on H_2_O_2_ and O_2_^−^ as key biomarkers of ROS, quantifying H_2_O_2_ and O_2_^−^ levels in wild-type and transgenic Arabidopsis lines grown under normal or drought stress conditions. H_2_O_2_ and O_2_^−^ levels were considerably lower in the transgenic Arabidopsis lines compared to wild-type seedlings (**Figures 10B, C**). Also, the activities of the antioxidant enzymes POD, SOD, and CAT, which can protect cell membranes against damage caused by ROS accumulation, were all markedly elevated in the transgenic lines relative to those in the wild type (**Figures 10D–F**). This suggested that the heterologous expression of *SbNF-YA6* in Arabidopsis bolstered the plant antioxidant machinery, thereby mitigating ROS-induced damage under drought conditions. MDA content serves as an indicator of membrane lipid peroxidation, a measure of cellular oxidative stress. In agreement with our above hypothesis, MDA content was significantly lower in the transgenic Arabidopsis lines than in the wild type under drought stress (**Figure 10G**), indicative of less extensive oxidative damage in the transgenic lines. Furthermore, we assessed the contents of osmolytes, specifically soluble protein and proline, which revealed that both accumulated to significantly higher levels in the transgenic Arabidopsis lines compared to the wild type under drought stress (**Figures 10H, I**). This rise in osmolyte contents suggests that the expression of *SbNF-YA6* helps maintain cellular osmotic balance, enhancing plant drought tolerance. Phenotypic observations of adult wild-type and transgenic Arabidopsis plants under drought stress demonstrated that whereas wild-type plants show severe wilting after a 7-day water withdrawal period, the transgenic plants exhibited milder symptoms. After a subsequent 7-day rewatering period, some transgenic plants recovered, whereas most wild-type plants had died (**Figure 10J**). These observations indicate that the heterologous expression of *SbNF-YA6* confers improved drought tolerance to Arabidopsis.

### Effects of *SbNF-YA6* heterologous expression in Arabidopsis on drought stress response genes

**Figure 11:**
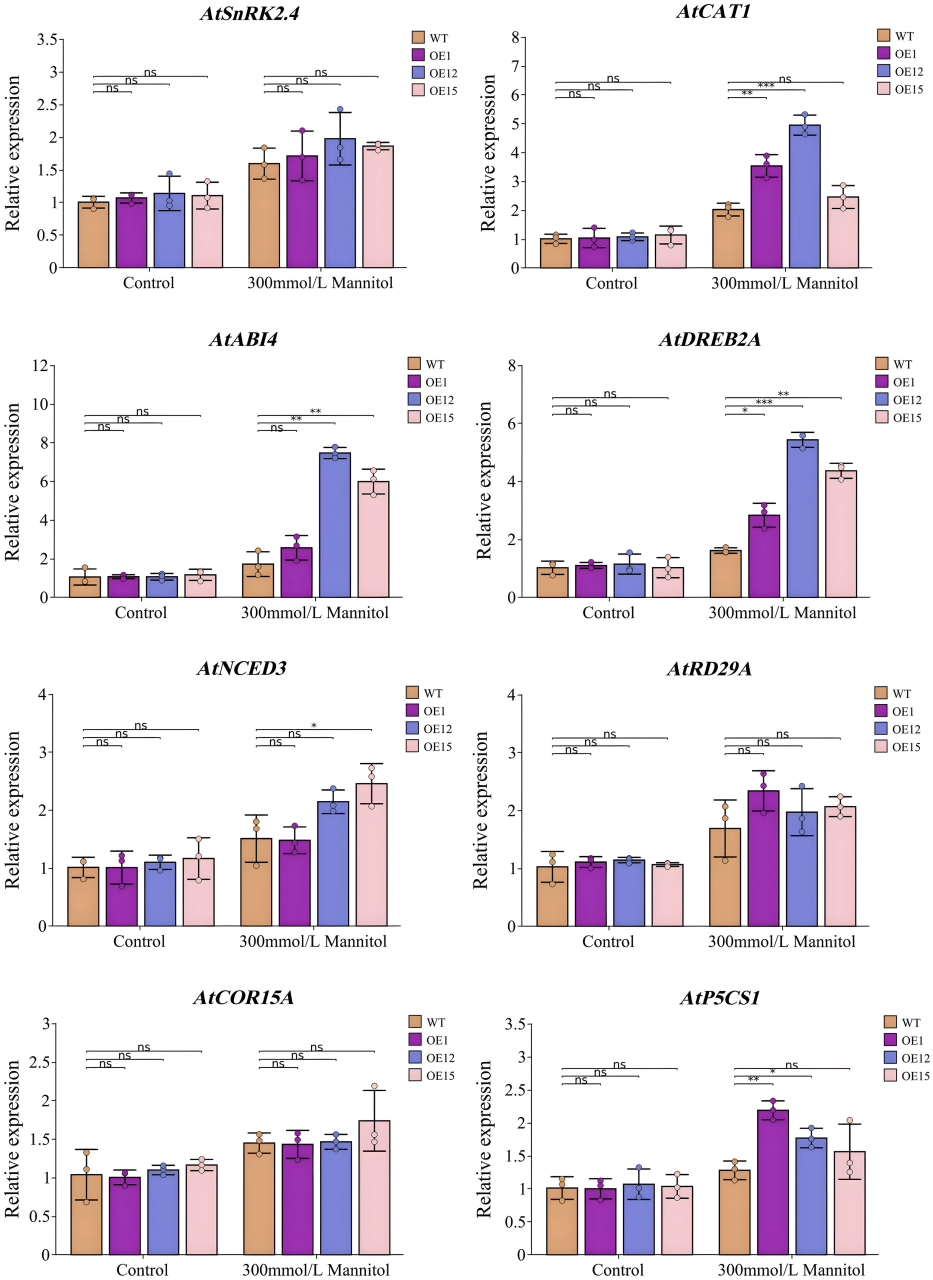
Expression analysis of drought stress response genes in Arabidopsis *SbNF-YA6*-OE lines. Seedlings were grown on MS medium without (Control) or with 300 mM mannitol to simulate drought stress. Values are means ± standard deviation from 3 technical repetitions independent experiments. Significant differences were determined by an independent two-sample *t*-test (ns, not significant, * *p* < 0.05, ** *p* < 0.01, *** *p* < 0.001).

To explore the molecular mechanism by which the heterologous expression of *SbNF-YA6* enhances drought tolerance in Arabidopsis, we conducted an RT-qPCR analysis of the expression levels of the drought stress-related genes *SNF1-RELATED PROTEIN KINASE 2.4* (*SnRK2.4*), *CAT1*, *ABA INSENSITIVE 4* (*ABI4*), *DREB2A*, *NINE-CIS-EPOXYCAROTENOID DIOXYGENASE 3* (*NCED3*), *RESPONSIVE TO DESICCATION 29A* (*RD29A*, *COLD-REGULATED 15A* (*COR15A*), and *DELTA1-PYRROLINE-5-CARBOXYLATE SYNTHASE 1* (*P5CS1*) in seedlings of the wild type and transgenic Arabidopsis lines (**Figure 11**). Under normal growth conditions, there was no significant difference in the transcript levels of the eight drought tolerance-related genes between wild-type and transgenic Arabidopsis lines. However, under drought stress conditions, the expression levels of all genes except *SnRK2*.*4*, *RD29A*, and *COR15A* were higher in the transgenic Arabidopsis than in the wild type, indicating that *SbNF-YA6* positively regulates some drought tolerance-related genes in Arabidopsis under drought stress, thereby enhancing drought tolerance.

## Discussion

### Structural characteristics and evolutionary relationship of the *SbNF-YA* gene family

The eukaryotic NF-Y transcription factor complex, composed of the three subunits NF-YA, NF-YB, and NF-YC, has essential roles in plant growth and development and in responses to biotic and abiotic stresses (Tian et al., 2024). The sequences of NF-YAs are highly evolutionarily conserved, but their functions are more diverse (Mu et al., 2013; Xing et al., 2022). NF-YAs can specifically recognize and bind to the CCAAT-box in the promoters of target genes to regulate their expression. With the rapid development of high-throughput sequencing, the NF-YA family members have been identified in various plant species, including Arabidopsis (Siefers et al., 2009), soybean (Quach et al., 2015), wheat (Stephenson et al., 2007), grape (*Vitis vinifera*) (Ren et al., 2016), and maize (Lv et al., 2022). The number of *NF-YA* genes varies among plant species (Jain et al., 2006). In this study, we identified nine *NF-YA* genes in the sorghum genome by bioinformatics analysis, unevenly distributed over four of the 10 sorghum chromosomes. Through a comprehensive analysis, we detected minimal variation among the NF-YA members between herbaceous and woody plants (Liu et al., 2022), corroborating prior findings.

A phylogenetic tree reconstructed based on the protein sequences of 77 NF-YAs from sorghum, *A. thaliana*, rice, maize, soybean, and foxtail millet showed that they were grouped into five clusters, with SbNF-YAs found in Clusters I, IV, and V and more closely related to the NF-YAs of maize and millet than to those of Arabidopsis and soybean. This finding is consistent with the closer overall evolutionary relationship among the monocot species sorghum, maize, and millet and their more distant relationship with the dicots Arabidopsis and soybean. This evolutionary pattern also suggests that some *NF-YA* members have emerged following the divergence between monocots and dicots (Chen et al., 2022). Furthermore, we determined that SbNF-YA4 resides in the same clade as ZmNF-YA1 (encoded by GRMZM2G000686_P01), which was previously shown to participate in root development and whose encoding gene responds at the transcriptional level to drought and salinity stresses (Yang et al., 2022). Therefore, we speculate that SbNF-YA4 has a biological function similar to that of ZmNF-YA1, offering clues for the functional characterization of SbNF-YA4.

The physicochemical properties of the nine SbNF-YAs revealed variations in their amino acid composition, molecular mass, and isoelectric point, potentially contributing to their functional diversity. SbNF-YA9 is the largest protein in the family of sorghum and is encoded by a gene with the highest number of exons (six). A longer DNA sequence in a locus gene is associated with a higher likelihood of mutations and breakpoints that may result in new proteins with unstable characteristics (Cao et al., 2023). SbNF-YA9 may therefore have followed a specific evolutionary trajectory. Most SbNF-YAs are predicted to localize in the nucleus, indicating that SbNF-YAs may mainly play a role in the nucleus, similar to results obtained from transient expression assays in *Nicotiana benthamiana* leaves (Tian et al., 2024). To elucidate the biological function of SbNF-YAs, we looked at the exon–intron structure of all *SbNF-YA* genes, finding that *SbNF-YA* genes in the same branch of the sorghum-restricted phylogenetic tree (**Figure 2**) show similar exon–intron structures. Most *SbNF-YA* genes contain five or six exons, aligning with the exon count observed for tomato *NF-YA* genes (Li et al., 2016), suggesting that the structure of *NF-YA* genes is relatively stable and evolutionarily conserved.

As with gene structure, members of the same subgroup share the same conserved motifs, while some motifs are present only in certain members, which may be related to their functional differentiation. Transcription factors generally exert their regulatory functions via conserved domains (He et al., 2022). The core domain alignment analysis showed that, notably, all nine SbNF-YA members possessed the core CBFB_NFYA domain consisting of an NF-YB/NF-YC interaction region and a DNA-binding domain, confirming their classification within the NF-YA family. Gene duplication events often occur among gene families, resulting in the specificity and diversity of gene functions, a major driving force of plant evolution (Ren et al., 2018). Genome colinearity analysis is an effective method to study the degree of evolution within and between species and whether gene pairs resulted from duplication events (Cao et al., 2023). We identified six *SbNF-YA* genes that have arisen from dispersed duplication events, indicating that such events have substantially contributed to the expansion of the *SbNF-YA* gene family. Furthermore, such genes may also undergo structural changes during replication that may alter their function. For example, some genes may still function as before after replication or may only acquire part of the function of the previous gene and may even lose function and become pseudogenes. We conducted a colinearity analysis between *SbNF-YA* genes and *NF-YA* genes from other plant species by constructing colinearity maps between the genomes of sorghum and those of Arabidopsis, maize, foxtail millet, and rice. We detected two *SbNF-YA* genes (*SbNF-YA4* and *SbNF-YA6*) as being colinear with their corresponding orthologs in the other four plant species, indicating that they may have existed before these species differentiated. Furthermore, seven *NF-YA* genes (*SbNF-YA1*, *SbNF-YA2*, *SbNF-YA4*, *SbNF-YA6*, *SbNF-YA7*, *SbNF-YA8*, and *SbNF-YA9*) were colinear with their orthologs in maize, foxtail millet, and rice. This finding suggests that these genes may have originated from common ancestral genes and have remained highly conserved throughout evolution. Similar NF-YAs in different species may have similar biological functions or participate in the same regulatory pathways. The high similarity and timeliness differences between similar gene sequences of various species provide infinite possibilities for the evolution and differentiation of species (Cao et al., 2023).

Small non-coding RNAs (miRNAs) play important roles in regulating gene expression in eukaryotes (Niu et al., 2021). We determined that seven miRNAs target the transcripts of four *SbNF-YA* genes. In Arabidopsis, miR169 targets the transcripts of several *NF-YA* genes to modulate the nitrogen response (Zhao et al., 2011; Yu et al., 2018). Overexpression of miR169a in Arabidopsis diminished the transcript levels of NF-YAs and the amount of nitrogen accumulated in plants (Zhao et al., 2011). Overexpression of miR169 in rice had the same effect on *OsNF-YA* transcripts and promoted rice growth under low-nitrogen conditions (Yu et al., 2018). While the regulation of *NF-YA* genes by miR169 has been explored, further investigation is required to elucidate the influence of other miRNAs on *NF-YA* expression. An analysis of the PPI network among SbNY-YAs showed that six of the nine SbNF-YA family members interact with one another, suggesting that the proteins regulate gene expression through the formation of heterodimers or heterotrimers.

### Response of *SbNF-YA* expression to plant growth and development and drought stress

Gene expression is regulated by *cis*-acting elements in promoters (Hernandez-Garcia and Finer, 2014). Therefore, we predicted the potential regulatory elements in the 2,000-bp promoter region upstream of the transcription start site of all *SbNF-YA* genes. The promoters of most *SbNF-YA* genes contain functional elements related to plant growth and development, phytohormone response, and stress response, indicative of probable roles for the genes in plant growth and development, phytohormone regulation, and stress responses. Gene expression patterns can to some extent reflect gene function (Xiao et al., 2019). Among *NF-YA* genes, several *NtNF-YA* genes in tobacco (*Nicotiana tabacum*) and *CmNF-YA* genes in melon (*Cucumis melo*) are highly expressed in roots (Tian et al., 2024; Li et al., 2023), while most *CsNF-YA* genes are more highly expressed in callus than in leaves (Pereira et al., 2018). We established here that the *SbNF-YA* genes are highly expressed in embryos, floral meristems, and vegetative meristems, suggesting that these genes regulate plant growth under adverse conditions and demonstrating that they have distinct expression patterns. During the past decade, *NF-Y* genes have been extensively studied as regulators of plant drought tolerance (Zanetti et al., 2017). Previous studies have shown that overexpression of *NF-Y* genes in Arabidopsis can enhance their drought tolerance (Yu et al., 2020). Moreover, the Arabidopsis genes *NF-YA1*, *NF-YA5*, and *NF-YA7* are involved in plant drought tolerance (Li et al., 2008, Li et al., 2013).

Similarly, the heterologous expression of *GmNF-YA3* or *GmNF-YA10* in Arabidopsis can minimize leaf water loss and improve plant drought tolerance (Yu et al., 2020; Ni et al., 2013). In this study, our analysis of published RNA-seq data showed that the expression levels of all *SbNF-YA* genes except those of *SbNF-YA5* responded to drought stress. Our independent validation by RT-qPCR showed that the expression levels of *SbNF-YA1*, *SbNF-YA4*, *SbNF-YA6*, *SbNF-YA8*, and *SbNF-YA9* were significantly upregulated upon drought stress, suggesting that these genes play a role in the drought response of sorghum. Similarly, *ZmNF-YA1* and *ZmNF-YA3*, which are homologous to *SbNF-YA4* and *SbNF-YA6*, respectively, encode proteins that have been shown to improve maize drought tolerance (Yang et al., 2022; Su et al., 2018). Therefore, we speculate that *SbNF-YA4* and *SbNF-YA6* contribute to sorghum drought tolerance.

### Heterologous expression of *SbNF-YA6* enhances the drought tolerance of transgenic Arabidopsis

The ABA signaling pathway is important for plant responses to drought stress (Zong et al., 2016). NF-Ys play important regulatory roles in the ABA signaling pathway, including regulating ABA biosynthesis and responses to ABA signaling and promoting auxin transport in roots to improve root growth (Xuanyuan et al., 2017). When Li et al. (2021) overexpressed *StNF-YC9* in potatoes and subjected the transgenic plants to drought stress, the transgenic plants had longer roots than wild-type control plants, while the photosynthetic rate of the transgenic plants was higher and the degree of water loss lower than in wild type. In this study, the heterologous expression of *SbNF-YA6* in Arabidopsis resulted in a higher germination rate compared to the wild type under simulated drought stress imposed by mannitol, along with significantly longer roots and greater fresh weight.

Drought stress raises ROS contents in plants and causes damage to plant cells. During drought stress, a series of antioxidant enzymes [such as SOD, POD, CAT, and ascorbate peroxidase (APX)] can scavenge ROS, thereby maintaining cell function and metabolic activity (Cao et al., 2023). In this study, we measured antioxidant enzyme activities in the wild-type and transgenic Arabidopsis *SbNF-YA6*-OE lines under normal conditions and drought stress, which showed that the SOD, POD, and CAT activities of the transgenic Arabidopsis lines were higher than in the wild type. Additionally, the transgenic Arabidopsis lines exhibited significantly lower rates of O_2_^−^ production and H_2_O_2_ content compared to those in the wild type, indicating an enhanced ability to mitigate excessive free radical accumulation by raising antioxidant enzyme activity under drought stress, thus minimizing drought-induced damage.

In addition to producing antioxidant enzymes to scavenge free radicals, plants also accumulate osmolytes to enhance their water retention capacity. Proline is an important osmotic adjustment substance that plays an active role in ROS scavenging and maintaining the stability of proteins, DNA, and cell membranes to resist oxidative damage caused by drought (Forlani et al., 2019). Soluble protein is also an essential osmotic adjustment substance and nutrient. Under drought stress, greater accumulation of soluble protein can improve the water retention capacity of cells and protect cell membranes. The transgenic Arabidopsis *SbNF-YA6*-OE lines accumulated elevated levels of proline and soluble protein compared to the wild type, suggesting an enhanced ability to synthesize osmotic regulators that maintain cellular membrane stability under drought stress, thereby mitigating damage. Overexpressing *ZmNF-YA1* in maize raised the content of osmotic adjustment substances in leaves, thus helping maintain a higher relative water content and significantly increasing antioxidant enzyme activity, thereby improving the free radical scavenging rate, lowering cell damage, and improving drought tolerance (Yang et al., 2022). Our results here are consistent with this study.

*SbNF-YA6* is a transcriptional activator. We hypothesize that under drought conditions, *SbNF-YA6* directly or indirectly modulates the expression of downstream target genes, including those involved in antioxidant enzyme activity, enzyme degradation, and proline biosynthesis, to sustain cellular osmotic potential, activate the antioxidant enzyme system, protect the cell membrane, and enhance drought tolerance in plants. Previous studies have shown that *ZmNF-YA1* can directly regulate the expression levels of *BASIC HELIX-LOOP-HELIX 116* (*ZmbHLH116*), *ZmPOD64*, *Α* (*ZmAMY5*), *CELL NUMBER REGULATE 9* (*ZmCNR9*), *LIPOXYGENASE 5* (*ZmLOX5*), *ZmMBF1c*, and *LESION SIMULATING DISEASE 1* (*ZmLSD1*) and enhance maize tolerance of abiotic stresses by binding to the CCAAT-box in the promoter regions of these genes (Yang et al., 2022). *ZmNF-YA3* binds to upstream bHLHs and basic leucine zippers (bZIP) through ABA-related pathways to respond to abiotic stress (Su et al., 2018). In this study, the expression of *NCED3*, a gene related to ABA biosynthesis, was significantly higher in the transgenic Arabidopsis lines than in the wild type under drought stress, indicating that *SbNF-YA6* affects the drought stress response by regulating ABA biosynthesis, at least when heterologously expressed in Arabidopsis. Furthermore, the expression levels of *CAT1* and *P5CS1* were significantly higher in transgenic Arabidopsis lines under drought stress compared to the wild type. *CAT1*, a key scavenger of H_2_O_2_, plays a crucial role in plant acclimation to drought stress. We thus conclude that *SbNF-YA6* induces the expression of *CAT1* and *P5CS1* to improve ROS scavenging ability, thereby enhancing the drought tolerance of Arabidopsis. At the same time, *SbNF-YA6* also induced the expression of the drought-tolerant genes *ABI4* and *DREB2A*. These results show that the heterologous expression of *SbNF-YA6* affects the transcript levels of drought stress-related genes in Arabidopsis and improves drought tolerance.

## Conclusion

In this study, we identified nine *NF-YA* family members in sorghum, with an uneven distribution on four out of the 10 sorghum chromosomes. We analyzed the gene structure, phylogeny, evolution, protein interaction, and expression characteristics of these *NF-YA* genes to reveal their structural characteristics, possible evolutionary mechanisms, and potential functions. Based on an analysis of transcriptome datasets, we speculate that *SbNF-YA1*, *SbNF-YA4*, *SbNF-YA6*, *SbNF-YA8*, and *SbNF-YA9* may play roles in the response to drought stress. The heterologous expression of *SbNF-YA6* in Arabidopsis improved the germination rate, root length, fresh weight, POD activity, SOD activity, CAT activity, soluble protein content, and proline content of transgenic Arabidopsis lines compared to the wild type under drought stress while decreasing the contents of H_2_O_2_, O_2_^−^, and MDA. The expression levels of drought tolerance-related genes such as *CAT1*, *ABI4*, *DREB2A*, *NCED3*, and *P5CS1* were also higher in the transgenic Arabidopsis *SbNF-YA6* lines than in the wild type, indicating that the overexpression of *SbNF-YA6* enhanced drought tolerance in Arabidopsis.

## Data Availability

The raw data supporting the conclusions of this article will be made available by the authors, without undue reservation.
