# Supplementary figures and images for: Identification of nuclear factor YA6 genes in sorghum and characterization of their involvement in drought tolerance
Source: Front Plant Sci. 2025 Mar 19;16:1524066. doi: 10.3389/fpls.2025.1524066 (PMC11961913; doi:10.3389/fpls.2025.1524066)

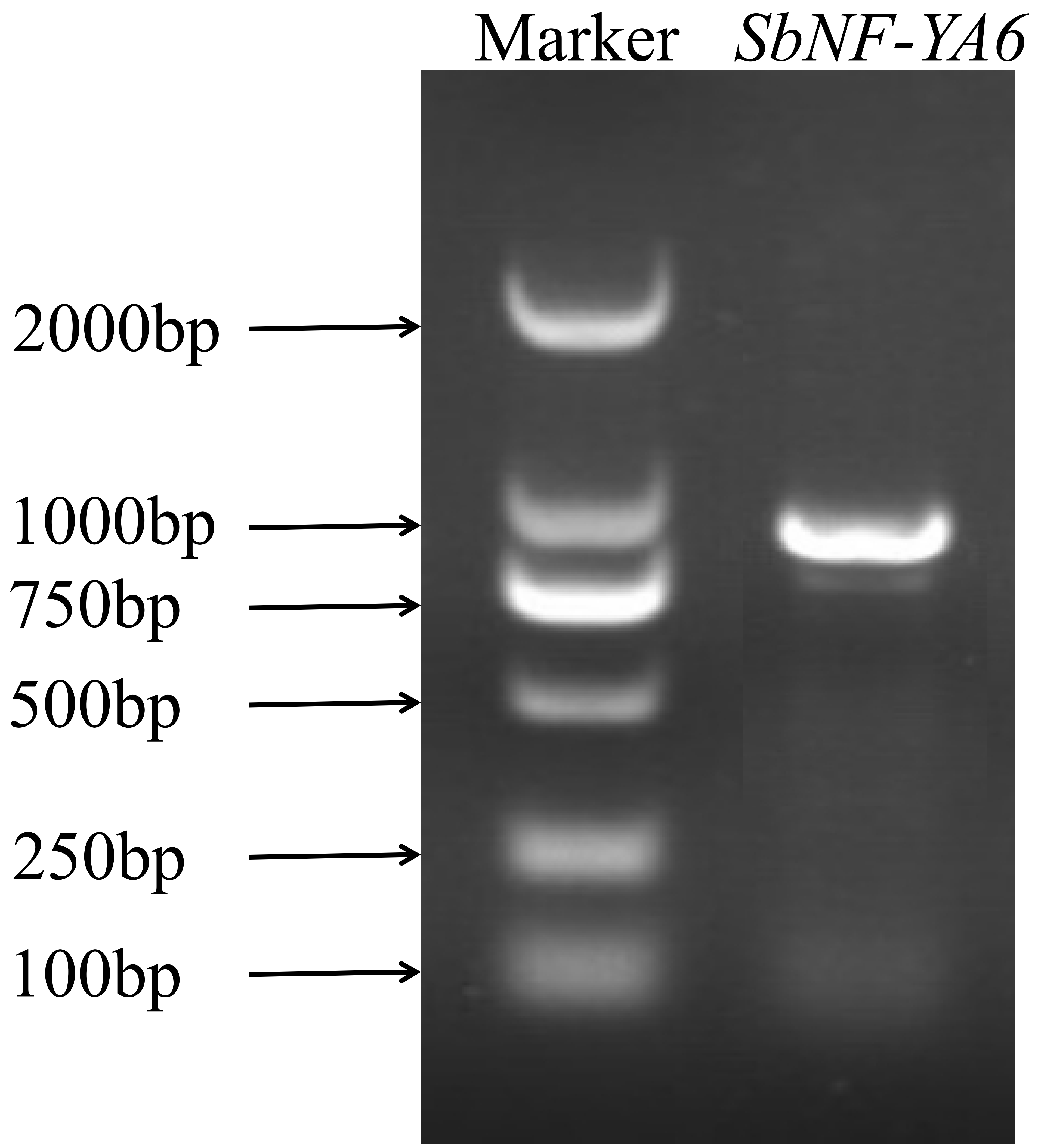

Supplement: Supplementary file 1 [file Image1.png]
