# Supplementary material for: Identification of nuclear factor YA6 genes in sorghum and characterization of their involvement in drought tolerance
Source: Front Plant Sci. 2025 Mar 19;16:1524066. doi: 10.3389/fpls.2025.1524066 (PMC11961913; doi:10.3389/fpls.2025.1524066)
Supplement: Supplementary file 2 [file Table1.docx]

**Supplementary Table S1** Abbreviations of this study

| bp | Base pair |
| --- | --- |
| qRT-PCR | Real-time fluorescent quantitative PCR |
| Kana | Kanamycin |
| ROS | Reactive oxygen species |
| POD | Peroxidase |
| SOD | Superoxide dismutase |
| CAT | Catalase |
| H_2_O_2_ | Hydrogen peroxide |
| O_2_^-^ | Superoxide anion |
| ABA | Abscisic acid |
| MDA | Malon dialdehyde |
| MeJA | Methyl jasmonate |
| DAB | 3,3’-Diaminobenzidine(DAB) Tetrahydrochloride |
| NBT | Nitrotetrazolium Blue chloride |
| HMM | Hidden Markov model |
| NJ | Neighbor-Joining |
| PPI | Protein-protein interaction |
