# Supplementary material for: Identification of nuclear factor YA6 genes in sorghum and characterization of their involvement in drought tolerance
Source: Front Plant Sci. 2025 Mar 19;16:1524066. doi: 10.3389/fpls.2025.1524066 (PMC11961913; doi:10.3389/fpls.2025.1524066)
Supplement: Supplementary file 3 [file Table2.docx]

Supplementary Table 2 List of primers used in this study

| Gene name | Primer sequence（5‘～3’） | | Purpose |
| --- | --- | --- | --- |
|  | Forward primer | Reverse primer |  |
| *SbEIF4a* | CAACTTTGTCACCCGCGATGA | TCCAGAAACCTTAGCAGCCCA | qPCR |
| *SbNF-YA1* | CCGAGGAGCCTGTTTATGTAAATGC | GAAGGTATGGCTTTCTGGCTTTGAC | qPCR |
| *SbNF-YA2* | CAGCACCATCACGCCTCACC | TGACACATCGGAGCCAGACAAG | qPCR |
| *SbNF-YA3* | ACAACGCACACCAACACAATCTG | GGGCTACCAAAGGACAACAAAGAC | qPCR |
| *SbNF-YA4* | TGCTCCCTCCTCATCTCACAGAG | TCCTTCGCTCATTCCAGACACTTC | qPCR |
| *SbNF-YA5* | CAGCAATAAGGCGAAGGCGAAG | CACCAGCGACGAGAGGAACAG | qPCR |
| *SbNF-YA6* | TAAGATGGTGAAAGGTCGGAAGCC | GGTACTGCTGGTTCTGCTCCTG | qPCR |
| *SbNF-YA7* | TTCCAGCAGCAACAGCAGTCC | CCACCACCAAGCCGTAGATGAG | qPCR |
| *SbNF-YA8* | TTATGCGGCTTACGGTGGACAG | AGGCTCTTCGATTGCATCAGTAGG | qPCR |
| *SbNF-YA9* | GCTGGGTGGTGGTTCGGTTAG | CGTGGGTGGTGCTCCTGAAG | qPCR |
| *AtActin8* | CTCAGGTATTGCAGACCGTATGAG | CTGGACCTGCTTCATCATACTCTG | qPCR |
| *AtCOR15A* | GGCCACAAAGAAAGCTTCAG | CTTGTTTGCGGCTTCTTTTC | qPCR |
| *AtP5CS1* | GCGCATAGTTTCTGATGCAA | TGCAACTTCGTGATCCTCTG | qPCR |
| *AtRD29A* | TGGACACGAATTCTCCATCA | TTCCAGCTCAGCTCCTGATT | qPCR |
| *AtNCED3* | ACCAACAAGAATGCCTTCCA | TAACAGAAACCAGCTGAGCTCGA | qPCR |
| *AtDREB2A* | GACCTAAATGGCGACGATGT | TCGAGCTGAAACGGAGGTAT | qPCR |
| *AtABI4* | AATCCGATTCCACCACCGAC | AGGGATACCGTACGGACCAA | qPCR |
| *AtCAT1* | CGCCATGCCGAAAAATACCC | CTTGCCTGTCTGAATCCCAGGAC | qPCR |
| *AtSnRK2.4* | GAGGAAATGGGGATGCAGAT | TTCTCACTTCTCCACTTGCG | qPCR |
| *R1f/r* | ATGCCTGTGCTTCTACGGGAA | TCACCTTATGGTGGAAACACGC | PCR |
| *R2f/r* | GACGCACAATCCCACTATCC | GGGTGAGCTTGCCGTAGGTG | PCR |
| *R3f/r* | GACGCACAATCCCACTATCC | TCACCTTATGGTGGAAAC | PCR |
